# Transcriptome Profiling of Trabecular Meshwork Progenitor Cells

**DOI:** 10.1007/s12015-025-10900-0

**Published:** 2025-05-27

**Authors:** Xiaochen Fan, Stephanie Kennedy, Emine K Bilir, Brian Lane, Olivia A Kingston, Xu Chen, Victoria R Kearns, Colin E Willoughby, Carl M Sheridan

**Affiliations:** 1 Department of Eye and Vision Science, Institute of Life Course and Medical Sciences, University of Liverpool, Liverpool, United Kingdom; 2 Department of Medicine, University of Cambridge, Cambridge, United Kingdom; 3 Genomic Medicine, Biomedical Sciences Research Institute, Ulster University, Coleraine, United Kingdom

**Keywords:** Trabecular Meshwork, Progenitor Cells, RNA-Seq, Primary open-angle glaucoma, Cell Differentiation

## Abstract

The loss and dysfunction of trabecular meshwork (TM) cells are implicated in aging and primary open-angle glaucoma. TM progenitor cells (TMPCs) contribute to the population and function of the TM, but their identity is not well elucidated. This study aimed to identify the expression profile of differentially expressed genes (DEGs) in human TM cell cultures, TM-derived spheres, and their differentiated progeny. Primary normal human TM cells (PTM) from three donors were cultured, de-differentiated into spheres, and re-differentiated into TM cells (DTM). RNA-Seq was performed using Illumina NGS, and bioinformatics analysis was conducted with Tuxedo, Bowtie2, Tophat, Cufflinks, and Ingenuity Pathway Analysis (IPA). DEGs were validated via Nanostring, RT-qPCR (in five independent donors), immunocytochemistry, and western blotting. RNA-seq identified significant DEGs in PTM, TM progenitor cells (TMPCs), and DTM cells. Gene expression in TMPCs differed significantly from PTM and DTM cells. Nanostring and RT-qPCR confirmed 70 DEGs upregulated in TMPCs (*P* < 0.05). Immunocytochemistry highlighted distinct markers in TMPCs (SOX2, NOTCH1, ANKG, MGP) versus PTM and DTM cells (TAGLN, TEM7, SPARC). Western blotting further analyzed MGP, TAGLN, and SPARC proteins, revealing significant upregulation of MGP in TMPCs and downregulation of TAGLN and SPARC in spheres compared to PTM cells. Pathway analysis revealed activation of cell cycle checkpoint regulation, SUMOylation, and STAT3 pathways in TMPCs, with HGF, MMP9, KDR, IGF1, and FOS as key node genes in TMPC development. RNA-Seq identified novel expression profile of potential TM markers and activated pathways in TMPCs, providing insights into TMPC behaviours in physiological and pathological conditions.

**Graphical Abstract:**

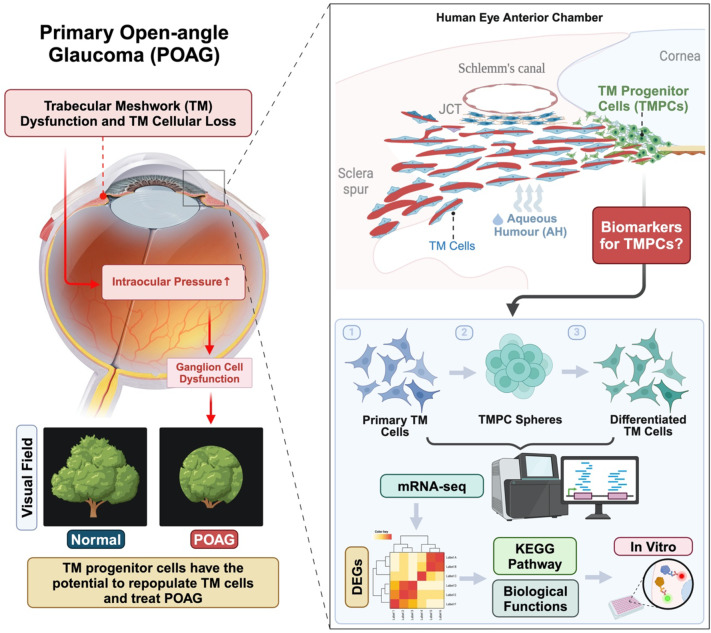

**Supplementary Information:**

The online version contains supplementary material available at 10.1007/s12015-025-10900-0.

## Introduction

Glaucoma is a leading cause of age-related blindness, affecting over 70 million people worldwide. The incidence of the disease is expected to rise as the population ages [1]. Currently, the only treatable risk factor for glaucoma is the reduction of elevated intraocular pressure (IOP). Studies have demonstrated that lowering IOP effectively slows disease progression in both early and advanced stages of glaucoma [2, 3].The balance between production and drainage of the aqueous humour (AH) from the anterior portion of the eye gives rise to the IOP. AH leaves the eye primarily through the trabecular meshwork (TM) before entering Schlemm’s canal, collector channels, and aqueous veins and collectively constitutes the major outflow pathways in the eye.

The TM, including the inner wall of Schlemm’s canal, is known to be the major site of aqueous outflow resistance, and changes in this tissue lead to elevated IOP. TM function is vital to AH outflow as the TM actively engulfs debris by phagocytosis [4, 5] and contributes to the contractility of the tissue. TM dysfunction and cell loss are all markedly increased with age and even more so with Glaucoma [6, 7]. Thus, replacing these TM cells represents a realistic therapeutic strategy. Whilst the resident TM cells have a limited regenerative capacity and they do not repopulate in vivo [7], it is known that surgical laser treatments can stimulate TM proliferation. Moreover TM cells can readily be cultured in vitro indicating their reparative potential [8]. Recent studies have shown that progenitors/stem cells of the trabecular meshwork cells exist [9–13]. Sphere culture of TM cells have demonstrated a population of progenitors cells can be readily obtained that can be directed towards functional differentiated TM [10].

Whilst, a combination of genes that are highly expressed in the TM have been assigned to characterise TM cells (including MYOCILIN [14], aquaporin-1 (AQP1) [15], matrix GLA protein (MGP) [16], chitinase 3-like 1 (CHI3L1) [17], alpha 2 A adrenergic receptors [18], alpha-B crystallin and collagen IV [19], there is still no definitive marker to identify TM progenitor cells. Indeed it is currently unknown whether there exists more than one progenitor exists as both progenitors of endothelium and trabecular meshwork (PET) [10] and MSC-TM [20] cell have been isolated from the insert region in bovine and porcine cornea as well as from human TM tissue.

This study defined the differentially expressed genes using Genome-wide transcriptome profiling (RNA-seq analysis) of human trabecular meshwork (TM) cell cultures, TM-derived spheres and their differentiated progeny. Additionally, it predicted key pathways and biological functions in TM-derived spheres and identified essential hub genes that regulate their development and maintenance.

## Methods

### Tissue Samples

Donor human corneal scleral tissues from de-identified cadaver donors (Table 1) were obtained from St Paul’s Eye Unit, Royal Liverpool University Hospital; UK, following use in surgery and handled in accordance with the tenets of the Declaration of Helsinki with Local Ethical approval (RETH000833). Donors between the ages of 21 and 85 years were used and had no clinical record of chronic illness or transmittable disease were used in experiments.

**Table 1:** The information of human donor eyes used in the experiment

Donor ID	Age	Eye Disease
HCR013	85	N
HCR020	75	N
HCR030	21	N
HCR050	28	N
NHS038	81	N
NHS040	88	N
IEB022	59	N
LREB046	88	N

Table 1 shows the donor ID and age of the human donor eyes used in the experiment. The donor HCR13, HCR020 and HCR030 were used for RNA-seq analysis. The donor NHS038, HCR050, IEB022, LREB046 and NHS040 were used for immunofluorescence, western blotting and RT-PCR analyses. N: no eye related disease

### Dissection of Trabecular Meshwork (TM) from Anterior Segment Human Eyes

Primary human TM cells were harvested from TM explants according to previously published methods [10, 21]. All tissue dissection was carried out under sterile conditions with an aseptic technique. The corneal rims were washed in phosphate-buffered saline (PBS) and stained with 0.1% trypan blue (Sigma, UK) to stain the TM and Scleral spur. The cornea was washed in PBS to remove excess trypan blue and extraocular tissue. The TM tissue was carefully dissected from the sclera using blunt forceps in a stripping technique using a dissection microscope. TM tissue was placed directly into incubating growth medium Dulbecco’s Modified Eagle’s Medium- low glucose (DMEM) (Sigma, UK) supplemented with 10% fetal Bovine serum (FBS) (Biosera, LabTech UK), 2 mM L-glutamine, 100 units’/ml 10% FBS). The strip of TM obtained was cut into sections of approximately 2–4 mm in length into a 6 well plate and was used thereafter to generate primary TM cells from explants cultures.

### TM Cell Culture

TM cells were cultured in growth medium, with the medium replaced every 2–3 days until 80–90% confluence was reached. For passaging, cells were dissociated using TrypLE™ Express Enzyme (Thermo Fisher, UK) for 5 min until complete detachment from the flask surface was observed. The TM cells were resuspended in sterile PBS and centrifuged at 1000 rpm for 5 min. The supernatant was discarded. The TM cell pellet resuspended in growth medium. TM cells were expanded up to passage 2–3 for use in experiments.

### Isolation and Propagation of TM Sphere

Following sub-culture of cells (passage 2 and 3), TM cell pellets were washed in sterile PBS to resuspend the pellet and centrifuged at 1000 rpm for 5 min. Following centrifugation cells were resuspended in serum and antibiotic free DMEM and cell number calculated. TM cells were resuspended in sphere culture medium at a cell density of 10000 cells/ml and seeded at a final cell density of 5000 cells per well (500 µl) into 24 well suspension plates (Greiner, UK) in sphere culture media (DMEM containing 1 X B27 (Invitrogen, UK), EGF 20 ng/ml (Peprotech, UK), bFGF 20ng/ml (Peprotech, UK) Heparin Sodium salt (5 µg/ml in 0.1% BSA (Sigma, UK)). Sphere cultures were supplemented every day with 10 X sphere culture media, with gentle pipetting of sphere culture solution up to 7 days. At 7 days, TM spheres were isolated by careful removal from wells using a 1 ml pipette tip and each well was washed in DMEM to collect any remaining spheres. Spheres were collected and centrifuged at 200 rpm for 5 min to settle the spheres and supernatant removed. Spheres were either re-suspended in TM cell growth media and cultured up to 7 days for differentiation of spheres onto tissue culture plastic or were processed for RNA extraction. TM cells at the same density were seeded into 24 well plates and for 7 days as controls. Mycoplasma testing was routinely carried out on conditioned media for all primary TM cells prior to experimentation with the Sigma LookOut ^®^ Mycoplasma PCR detection kit (Sigma, UK) according to manufacturers’ specifications. Preceding experimentation the TM cells were observed throughout their lifespan under standardised cell culture conditions. These cells were viewed under a Zeiss confocal or Nikon Brightfield microscope and images were taken were taken routinely throughout cell culture to monitor growth of the cells ensuring appropriate morphology was maintained.

### Characteristics of Trabecular Meshwork Cells in Vitro

The primary TM cells were routinely characterised by immunocytochemistry and western blotting to stain for the positive presence of myocilin. Briefly, the TM cells were cultured on glass chamber slides (Millipore, UK) or 6-well culture plates at a cell density 20,000 cells per cm^2^. The cells were serum starved for 24 h at 70% confluency and then treated with/without 100nM dexamethasone (DEX) every other day for 7 days. The TM cells were fixed or lysed for further immunocytochemistry or western blotting analysis of the myocilin expression.

#### Immunocytochemistry

Primary TM cells (pTM), TM spheres and differentiated TM cells (DTM) were fixed with 10% (v/v) neutral buffered formalin (NBF) (Sigma, UK) for 15 min following media removal and sterile PBS wash. Fixed cells were washed with PBS and permeabilised with 0.5% (v/v) Triton-X 100 (Sigma, UK)/PBS solution for 5 min. The cells were subsequently washed with PBS (3 × 5 min). To reduce non-specific binding, the cells were blocked in the blocking buffer (0.1% BSA, 0.2% Triton X-100 and 10% normal goat serum (Sigma, UK) in D-PBS). Following PBS wash (3 × 5 min), the cells were incubated in primary antibodies (Table 2) diluted in 1% (v/v) BSA/PBS overnight at 4^o^C. After overnight incubation the cells were washed thoroughly with 0.1% (v/v) Tween 20/PBS (PBS-T) wash buffer and incubated with Alexa Fluor conjugated secondary antibodies (Table 3) at assay dependent concentrations for 60 min at 37^o^C. The cells were then washed with PBS-T wash buffer (3 × 5 min). Nuclei were counterstained with 4′, 6-diamidino‐2‐phenylindole dihydrochloride (DAPI) (Invitrogen and diluted in methanol or deionized water following the manufacturer’s instructions). The cells were washed with PBS-T wash buffer (3 × 5 min) and mounted using Fluoroshield Mounting Medium (Abcam, UK). ZEISS LSM 800 microscope was used for imaging (Zeiss, UK).

**Table 2:** Information of the primary antibodies used for immunocytochemistry

Primary Antibodies	Company	Catalogue	Species	Dilution
Myocilin	Abcam	Ab55477	Mouse	1:50
CHI3L1	Abcam	ab77528	Rabbit	1:100
MGP	Thermo Fisher	MA526799	Mouse	1:100
SPARCL1	Abcam	ab107533	Rabbit	1:50
SOX2	Proteintech	20118-1-AP	Rabbit	1:50
PAX6	Proteintech	12323-1-AP	Rabbit	1:50
SPARC	Haemtech	AON-5031	Mouse	1:50
TAGLN	Thermo Fisher	PA513632	Rabbit	1:10
TEM7	Thermo Fisher	MA141065	Mouse	1:10
COL4 A1/2	Thermo Fisher	MA122148	Mouse	1:100
Nestin	Abcam	Ab105389	Rabbit	1:200
IgG mouse	Thermo Fisher	10,400 C	Mouse	1:600
IgG rabbit	Thermo Fisher	10,500 C	Rabbit	1:600

Table 2 shows the information of the antibodies used for immunocytochemistry included the antibody names, company, catalogue number, species and dilution information

**Table 3:** Information of the secondary antibodies used for immunocytochemistry

Secondary Antibodies	Company	Catalogue	Dilution
Goat anti-Rabbit, Alexa Fluor Plus 594	Thermo Fisher	A-11,012	1:500
Goat anti-mouse, Alexa Fluor Plus 594	Thermo Fisher	A-11,005	1:500
Goat anti-mouse, Alexa Fluor Plus 488	Thermo Fisher	A-11,001	1:500

Table 3 shows the information of the secondary antibodies used for the immunocytochemistry included the antibody names, company, catalogue number and dilution information

### Western Blotting

The cells were washed twice with ice-cold sterile PBS and lysed in ice-cold RIPA lysis buffer (catalogue: 89900, Thermo Fisher Scientific, UK) with a protease inhibitor cocktail (catalogue: 10658304, Thermo Fisher Scientific, UK) for 5 min. The cell lysates were harvested by scraping and transferred into pre-cooled micro-centrifuge tubes. The samples were sonicated for 30 s and centrifuged at 14,000xg for 15 min at 4 °C. The samples were then stored in a −80 °C freezer until use.

According to the manufacturer’s description, the quantity of the protein was analyzed using the Pierce Microplate BCA Protein Assay Kit (Thermo Scientific, UK). The sample (containing 20 µg proteins) were transferred to a new micro-centrifuge tube and mixed with an equal volume of 2X Laemmli sample buffer (1610737, Bio-Rad, UK). The samples were then boiled at 95 °C for 5 min and centrifuged at 16,000xg for 1 min.

The SDS-PAGE gel was prepared according to the molecular mass of the target proteins. The samples and Precision Plus Protein Dual Color Standards (Bio-RADD, UK) were loaded into the SDS-PAGE gel. The proteins were resolved by using the Mini-PROTEAN Tetra Handcast Systems (BIO-RAD, UK). The protein was then transferred to the Immun-Blot^®^ Low Fluorescence PVDF Membrane (BIO-RAD, UK) using the Mini-PROTEAN Tetra Handcast Systems at 100 V for 2 h. The membranes were blocked in Odyssey blocking buffer TBS (LI-COR, UK) for 1 h at room temperature and incubated with primary antibodies (Table 4) overnight at 4 °C. The membranes were then rinsed 3 times with 0.1% (v/v) Tween 20/tris-buffered saline (TBS-T) wash buffer and incubated with secondary antibodies (Table 5) (dilution 1:20,000; LI-COR, UK) for 1 h at room temperature. After washing with TBS-T wash buffer 3 times for 5 min, the membrane was analyzed by Odyssey imaging system (LI-COR, UK).

**Table 4:** Information of the primary antibodies used for Western blotting

Primary antibodies	Company	Catalogue	Species	Dilution
Myocilin	Abcam	Ab55477	Mouse	1:50
MGP	Thermo Fisher	MA526799	Mouse	1:100
SPARC	Haemtech	AON-5031	Mouse	1:50
TAGLN	Thermo Fisher	PA513632	Rabbit	1:10

Show Information of the secondary antibodies used for western blottings the information of the primary antibodies used for the Western blotting included the antibody names, company, catalogue numbers, species and Dilution information

**Table 5:** Information of the secondary antibodies used for western blotting

Secondary antibodies	Company	Catalog	Dilution
IRDye 680RD Goat anti-Rabbit	LI-COR	C80816-15	1:15000
IRDye 800 CW Goat anti-Mouse	LI-COR	C80605-15	1:15000

Table 5 shows the information of the secondary antibodies used for western blotting included the name, company, catalogue number and dilution information

### Ribonucleic Acid (RNA) Extraction

Total RNA was extracted using AllPrep^®^ DNA/RNA/miRNA Universal Kit (Qiagen, UK) as per manufacturers’ specifications. Briefly, pTM and DTM cells grown in monolayers and TM spheres were lysed with 350 µl RLT lysis buffer. The lysate was homogenized by transferred to a QIAshredder column (Qiagen, UK) and centrifuge for 2 min at maximum speed. On column DNase I digestion (Qiagen, UK) was carried out prior to subsequent steps according to the manufacturer’s instructions. RNA (including miRNAs) was eluted in with 30µL of RNase-free water and a second elution with 20 µL RNase-free water RNA quality and yield was analyzed using the Agilent RNA 6000 Nano kit on the Agilent 2100 Bioanalyser (Agilent Technologies, UK), prior to long term storage at −80^o^C until experimental use. RNA samples with an RNA Integrity Number (RIN) above 9 and 50 ng/µl were used for sequencing experiments.

### RNA-Seq and MicroArray Analyses

#### Exiqon RNA-Seq Analysis

RNA was obtained from pTM cells, spheres and DTM cells of three individual donors (HCR13, HCR020 and HCR030). RNA-Seq experiments were performed by Exiqon (Exiqon, Denmark). Following RNA-Seq intensity correction and base calling (into BCL files), FASTQ files were generated using appropriate bcl2fastq software (Illumina Inc, UK), which included quality scoring of each individual base in a read. Subsequently, data was separated for paired-end reads to determine whether the second read significantly differs from the first in terms of overall quality.

Data analysis was initially performed by Exiqon (Exiqon, Denmark). The components of Exiqon Launches Next Generation Sequencing (NGS) RNA-Seq analysis pipeline includes Bowtie2 (v2.2.2), Tophat (v.2.0.11) and Cufflinks (v2.2.1). Tophat is a fast splice junction mapper for RNA-Seq reads. Tophat was used to align the sequencing reads to the reference genome (h.sapiens, hg19/GRC37, UCSC Genome Browser) using the sequence aligner Bowtie2. Tophat was also utilized to identify splice junctions for both known and novel transcripts from the sequence alignments.

Cufflinks received alignment results from Tophat and was used to assemble the aligned sequences into transcripts, this constructing a map of the transcriptome. To guide the assembly process, an existing transcript annotation was used. In addition, Exiqon performed fragment bias correction which sought to correct for sequence bias during library preparation. Cufflinks then assembled aligned reads into different transcript isoforms based on exon usage and also determined the transcriptional start sites (TSSs).

It is necessary to build linear model groups with samples paired by the donor eye. The limma functionality of edgeR is ideal for this but requires counts of mapped reads as an input. Counts were generated using feature Counts and inference of differential expression was by generalized linear model likelihood ratio test (glmLRT) implemented by edgeR.

#### Bioinfokit Toolkit/Python

The normalized data and DEGs data of the RNA-seq were uploaded into the Bioinfokit Toolkit (10.5281/zenodo.3698145) in Python (v 3.8.3) for generating the volcano plots and heat-map. The Log2 FC and P-value of the DEGs were used for generating the volcano plot. For generating the heat-map, the FKPM values of the top 100 DEGs (based on smallest P-value, P-value < 0.05) were transformed to In (FPKM + 1) and uploaded to the Bioinfokit Toolkit. The PCA plot was generated using the ClustVis tool [22].

#### Ingenuity Pathway Analysis (IPA)

The pathway and function analyses were performed by Ingenuity Pathway Analysis (IPA, QIAGEN Inc.,https://www.qiagenbioinformatics.com/products/ingenuitypathway-analysis). The DEGs data was uploaded into the IPA software. The core analysis function in IPA was used to analyse the pathways and biological functions. Significance (P-value) is calculated using a Right-Tailed Fisher’s Exact Test. *P* < 0.05 was considered to be statistically significant. The Z-score was calculated by IPA for providing the prediction of the activation or inhibition of the pathways and biological functions. The Z-score > 2 or <−2 is considered significant.

#### Cytoscape/String

For analysing the node genes that participated in the cell development-related functions in RNA-seq data, the LogFC and P–value of the top 50 DEGs (based on smallest *P*-value, *P*-value < 0.05) were uploaded in the String [23]. The generated network was then uploaded into the Cytoscape software [24]. The network analyser function was used for analyzing the node degree of each gene. The node gene networks were generated based on the degree of centrality.

#### Nanostring

The RNA from pTM cells, spheres and DTM cells were analyzed by using the Nanostring for confirming the part of the RNA-seq data. The Nanostring assay was performed by Centre for Genomic Research at the University of Liverpool. A total of 100ng mRNA was hybridized with the Fibrosis Panel (NanoString Technologies, USA) which included 770 genes involved in the fibrosis. The output data was imported into the nSolver 4.0 software (NanoString Technologies, USA) for sample quality control and data normalization. The normalized data was further imported into the cloud-based ROSALIND (Onramp Bioinformatics, USA) for analyzing the differentially expressed genes. The Nanostring DEGs were imported into the Ingenuity Pathway Analysis software (IPA, Qiagen, UK) for analyzing the canonical pathways. The Nanostring statistically significant DEGs and canonical pathways were compared with the RNA-seq DEGs and canonical pathways by using the automatic analysis algorithm-Python (v 3.8.3) for finding the consistent data.

#### cDNA Synthesized

Complementary DNA (cDNA) was synthesized from RNA extracted from pTM cells, spheres, and DTM cells using the Primer Design cDNA Synthesis Kit. A total of 100 ng of RNA was reverse-transcribed according to the manufacturer’s instructions. Briefly, the template RNA was combined with nuclease-free water and added to the reverse transcription master mix, which contained 5× HiFlex Buffer, 10× Nucleics Mix, and miScript Reverse Transcriptase, following the manufacturer’s protocol. The reaction mixture was incubated at 37 °C for 60 min, followed by enzyme inactivation at 90 °C for 5 min, using a Veriti 96-well Thermal Cycler (Applied Biosystems™, Thermo Fisher Scientific, UK).

#### Primer Design and Optimization

All primers were designed by Primerdesign Ltd (Primerdesign Ltd, UK) or sequences were obtained from Primer Bank website. (http://pga.mgh.harvard.edu/primerbank/*)* Hybridization of the primers to the desired section of DNA was verified using NCBI nucleotide BLAST (https://blast.ncbi.nlm.nih.gov/Blast.cgi?PAGE_TYPE=BlastSearch*).*

For each primer set, pairs were diluted to 1µM final concentration (10µL sense primer, 10 µL antisense primer and 80µL H_2_O). For each primer pair, a standard curve was prepared using 3-fold serial dilutions of a mixture of cDNA previously prepared. Dilutions were made for at least 5 concentrations ranging from 300 ng-4ng to ensure complete coverage of the range of expected gene expression. Each concentration was done in duplicate. The slope of the standard curve and q-PCR efficiency for each primer pair was determined. Primer pairs with an efficiency of 1.8–2 were accepted. Melt curve analyses were also employed to determine the efficiency of primer pairs. Glyceraldehyde-3-phosphate dehydrogenase (GAPDH) is a commonly used housekeeping gene for mRNA expression analyses [25] and is stably expressed in TM cells.

#### Relative Quantification real-time PCR (q-PCR)

Relative quantification (ΔΔCt method) real-time PCR was performed to investigate the expression of specific mRNA targets in samples. qPCR was performed on LightCycler ^®^ 96 (Roche, UK) according to the manufacturer’s instructions. For each 10 µl final volume of qRT-PCR, unlabeled gene-specific primers (Table 6) were diluted to 1µM final concentration and mixed with 1X PrecisionPLUS SYBR Green qPCR Master Mix (Primerdesign Ltd, UK). Each cDNA template was added triplicate in 1 µg final concentration.

**Table 6:** Information of the primers used for RT-qPCR

Gene ID	Forward primer sequence	Reverse primer sequence	Melting temp (°C)
Classical TM cell marker			
MGP	5’ GCCGCCTTAGCGGTAGTAAC 3’	5’ TCTCTGCTGAGGGGATATGA 3’	60
AQP1	5’ TAACCCTGCTCGGTCCCTTG 3’	5’ AGTCGTAGATGAGTACAGCCAG 3’	60
CHI3L1	5’ CCTTGACCGCTTCCTCTGTA 3’	5’ GTGTTGAGCATGCCGTAGAG 3’	60
Stem cell markers			
SOX2	5’ AACCAGCGCATGGACAGTTAC 3’	5’ TGGTCCTGCATCATGCTGTAG 3’	60
OCT4	5’ GAGAAGGATGTGGTCCGAG 3’	5’ TCCTCTCGTTGTGCATAGTC 3’	60
ANKG	5’ GAAGATGCAATGACCGGGGA 3’	5’ CTGAAGCCCATGTAACCCTCTG 3’	60
NES	5’ CAGAGGTGGGAAGATACGGT 3’	5’ AGCTCTGCCTCATCCTCATT 3’	60
NOTCH1	5’ AGTCTCTGCAGTGCTGGAAGTA 3’	5’ CTTGCAGTACTGGTCGTACAGG 3’	60
Potential DEGs			
TAGLIN	5’ AAGCCTTCTTTCCCCAGACA 3’	5’ TGCACTATGATCCACTCCACC 3’	60
SPARC	5’ CTGGACTACATCGGGCCTTG 3’	5’ ATGGATCTTCTTCACCCGCAG 3’	60
SPARCL1	5’ GCTGCAATCCCGACAAATGC 3’	5’ AGTGTTGTCAGGTGCTACCG 3’	60
COL4 A1	5’ GGACGCAGGACTCTGAACAT 3’	5’ CAGCCACCCTTCAACCGT 3’	60
COL4 A2	5’ TGTCTCCATCCCACACTGCC 3’	5’ CCTCTAGACAGCTGCCCGGT 3’	60
TEM7	5’ ACACGCTGCCAGATAACAG 3’	5’ TCGGCCACATCTACCCACA 3’	60
GAPDH	5’ CGAGCCACATCGCTCAGACACC 3’	5’ GGTCAATGAAGGGGTCATTGATGGCAAC 3’	60

Table 6 shows the information of the primers used for RT-qPCR included the primer sequences and melting temperature

### Statistical Analysis

All statistical analyses were conducted using GraphPad Prism version 8.0.2 (GraphPad Software, San Diego, CA, USA; http://www.graphpad.com/quickcalcs/ConfInterval1.cfm). For multiple group comparisons, one-way analysis of variance (ANOVA) was employed for parametric data, while the Friedman test or the Kruskal–Wallis test was used for non-parametric data, depending on the distribution and variance homogeneity assessed through the Shapiro–Wilk test and Levene’s test, respectively. Post hoc pairwise comparisons were performed using Tukey’s multiple comparisons test for ANOVA, Dunn’s test for Kruskal–Wallis, and the Wilcoxon signed-rank test for Friedman analysis. A *p*-value of less than 0.05 was considered statistically significant. Statistical significance was denoted as follows: *p < 0.05, **p < 0.01, ***p < 0.001, and ****p < 0.0001.

## Results

### RNA-Seq analysis of the gene expression of primary TM cells, TM spheres and differentiated TM cells

The mRNA of primary TM cells, TM spheres and differentiated TM cells from three donors (HCR013, HCR020, HCR030) were analysed by RNA-seq. The differentially expressed genes between three cell types were further analysed using IPA, bioinfokit toolkit/Python, ClustVis, String and Cytoscape RNA-seq analysis for comparing the gene expression, pathways and biological functions between three cell types.

#### Clustering and Visualization of the Differentially Expressed Genes

To determine the differentially expressed gene in PTM, spheres and DTM cells, the mRNAs from the three cell types were analysed by RNA-seq. A total of 6396 genes was found to be significantly differentially expressed in spheres and PTM cells (adjust-*P* < 0.05). A total of 5852 genes was differentially expressed in PTM and DTM and a total of genes were differentially expressed in the spheres and DTM cells (*P* < 0.05).

**Fig. 1:**
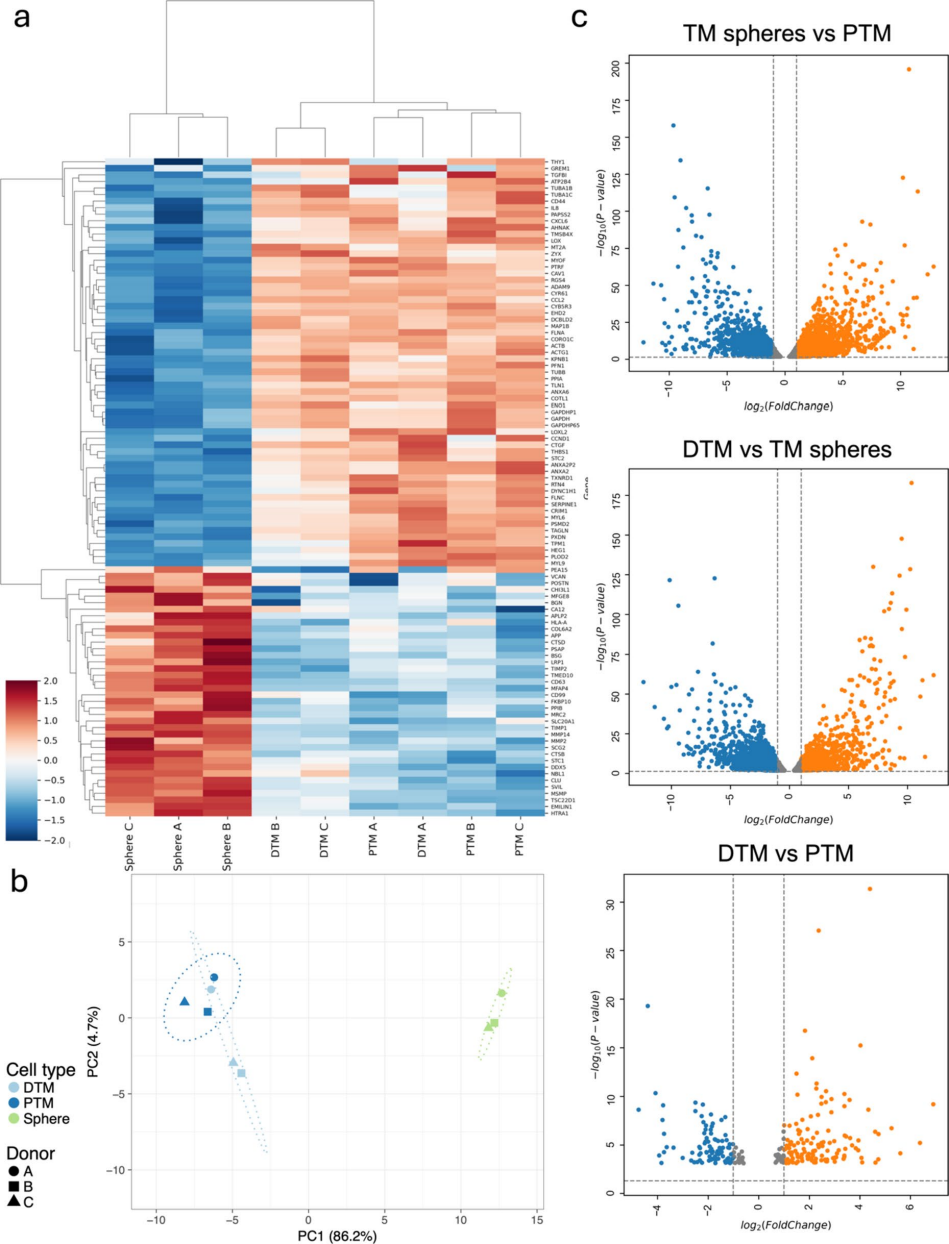
Shows visualization and clustering of the differentially expressed genes in the PTM, TM sphere and DTM cells analysed by RNA-seq. **a**: Heatmap of the gene expression in PTM, TM spheres and DTM cells. The red colour means the transformed FPKM value of a gene in a sample is higher than the mean of the transformed FPKM values in all the samples (Z score > 0). The blue colour means the transformed FPKM value of a gene in a sample is lower than the mean of the transformed FPKM values in all the samples (Z score < 0). **b**: Principal component analysis of the gene expression in PTM, TM spheres and DTM cells. In the PCA plot, different colours of the dots mean different cell types (Dark blue: PTM; light blue: DTM; green: TM spheres). The different shapes of the dots mean the different donors (round: donor A; square: donor B; triangle: donor C). **c**: Volcano plot of the DEGs in PTM, TM spheres and DTM cells; The volcano plot showed the DEGs in the three comparison groups (Sphere vs. PTM; sphere vs. DTM, and DTM vs. Sphere). Orange colour: the DEGs with log 2-fold change > 1.5 and *p*-value < 0.05; Blue colour: the DEGs with log 2-fold change<- 1.5 and *p*-value < 0.05. Grey dots: the excluded genes with the log2 FC>−1 and < 1, and *P*-value > 0.05

Heatmap was generated to show the expression of the top 50 DEGs (Sphere vs. PTM, based on the smallest *P*-value) in the three cell types from the three different donors (Fig. 1-a). The FPKM values of the top 50 DEGs were transformed to ln(FPKM + 1) and graphed by Bioinfokit Toolkit package/Python. In the heatmap, the rows represented different genes, and the columns represented different cell types (PTM cells, TM spheres and DTM cells) from different donors (A, B and C). The Z-score was used to standardize the transformed FPKM values (the standardization is for exploring the difference of the data in the same row of the heatmap).

PCA plot was generated to show the clusters of the three cell types (PTM, spheres and DTM cells) from three donors based on their similarity (Fig. 1-b). In the PCA plot, the different colours of the dots represent different cell types (TMPC sphere: green; PTM: dark blue; and DTM: light blue). The different shapes of the dots represent different donors. The PCA plot showed that the gene expression of PTM and DTM cells was similar, while the gene expression of TM spheres differed from the PTM and DTM cells.

Volcano plots were generated to enable visual identification of DEGs with fold change and statistically significant (Fig. 1-c). The volcano plots were constructed by plotting the–log10 (*p*-value) on the Y-axis and the log2 FC on the X-axis. A higher value of the Y-axis means a lower P-value. The orange dots showed the DEGs with log2 FC > 1.5 and–log10 (*p*-value) > 1.3. The blue dots showed the DEGs with log2 FC<−1.5 and–log10 (*p*-value) > 1.3. The volcano plots showed that the gene expression of TM spheres and PTM cells significantly differed. The gene expression of DTM cells was more similar to the PTM cells than the TM spheres.

#### Top 15 DEGs

The top 15 DEGs in three different comparison groups (sphere vs. PTM, DTM vs. sphere and DTM vs. PTM) based on the lowest *p*-values were listed in the Tables 7, 8 and 9. The tables included the gene names, LogFC and *p*-values of the top 15 DEGs. The whole DEGs in three groups were shown in the Online Resource-2.

**Table 7:** Top 15 Up-regulated DEGs in the sphere (vs. PTM/DTM)

Gene ID	Gene Name	Sphere vs. PTM		DTM vs. Sphere	
		LogFC	*P*-Value	LogFC	*P*-Value
LSP1	Lymphocyte Specific Protein 1	12.85201048	2.86E-63	−8.232547908	1.35E-53
PI15	Peptidase Inhibitor 15	12.3365877	4.95E-58	−12.35039253	3.48E-58
RASL12	RAS Like Family 12	11.47991226	5.37E-114	−9.394765553	4.24E-106
H19	H19 Imprinted Maternally Expressed Transcript	11.39680507	2.47E-42	−11.39999372	1.83E-42
CAMP	Protein Kinase cAMP-Dependent Type I Regulatory Subunit Alpha	11.13238989	4.41E-42	−9.278035942	2.22E-40
PLXDC1	Plexin Domain Containing 1	10.72664553	1.62E-196	−6.323728887	2.36E-123
AC005013.1	AC005013.1	10.68712524	8.10E-23	−6.621588284	3.97E-15
GABRR1	Gamma-Aminobutyric Acid Type A Receptor Subunit Rho1	10.5863879	5.43E-35	−10.62221856	4.45E-35
KCNJ6	Potassium Inwardly Rectifying Channel Subfamily J Member 6	10.52625703	1.35E-30	−6.34161302	5.83E-24
TRIL	TLR4 Interactor with Leucine Rich Repeats	10.35415494	1.27E-77	−7.728410938	1.18E-64
TRDC	T Cell Receptor Delta Constant	10.21659898	3.64E-30	−10.22704061	3.04E-30
COL15 A1	Collagen Type XV Alpha 1 Chain	10.19901883	2.75E-123	−10.13317383	3.36E-122
KDR	Kinase Insert Domain Receptor	10.17779995	3.98E-24	−7.11082689	1.25E-16
RP4-668G5.1	RP4-668G5.1	10.00277295	3.17E-31	−5.568293137	2.64E-23
HAPLN2	Hyaluronan And Proteoglycan Link Protein 2	9.418751217	1.89E-19	−9.351719209	1.71E-19

Table 7 shows the gene ID, gene name, Log FC and *P*-value of the top 15 up-regulated DEGs in the spheres (vs. PTM/DTM) based on the largest logFC value in Sphere vs. PTM group (P<0.05)

**Table 8:** Top 15 Down-regulated DEGs in the sphere (vs. PTM/DTM)

Gene ID	Gene Name	Sphere vs. PTM		DTM vs. Sphere	
		LogFC	*P*-Value	LogFC	*P*-Value
MYBL2	MYB Proto-Oncogene Like 2	−11.36054077	8.85E-52	12.22777566	1.55E-62
NEK2	NIMA Related Kinase 2	−10.69022122	1.10E-50	11.28196665	2.50E-59
SKA1	Spindle and Kinetochore Associated Complex Subunit 1	−10.43363009	2.96E-41	11.10065334	4.77E-49
PTPRQ	Protein Tyrosine Phosphatase Receptor Type Q	−10.3898142	5.22E-25	8.450906682	3.56E-15
RRM2	Ribonucleotide Reductase Regulatory Subunit M2	−9.630911966	1.36E-158	10.3590096	2.09E-183
BIRC5	Baculoviral IAP Repeat Containing 5	−9.526984546	4.69E-110	10.24284287	4.21E-129
RP11-818 F20.5	RP11-818 F20.5	−9.316907866	1.56E-33	8.123246101	6.96E-21
KIF18B	Kinesin Family Member 18B	−9.230366766	3.56E-63	9.806325602	5.19E-74
DLGAP5	DLG Associated Protein 5	−9.200135958	5.30E-88	9.921662658	1.37E-103
SLC28 A3	Solute Carrier Family 28 Member 3	−9.076295124	9.87E-23	7.955283238	1.28E-15
MKI67	Marker of Proliferation Ki-67	−9.018972001	4.94E-135	9.52067236	2.77E-148
UBE2 C	Ubiquitin Conjugating Enzyme E2 C	−8.773840003	3.58E-76	9.536485273	1.94E-91
RP11-456 J20.1	RP11-456 J20.1	−8.652036435	7.84E-23	9.373876105	2.93E-29
TK1	Thymidine Kinase 1	−8.531327507	7.21E-103	9.344761178	4.98E-125
CPA4	Carboxypeptidase A4	−8.359006428	3.66E-25	6.791986674	1.26E-18

Table 8 shows the gene ID, gene name, Log FC and *P*-value of the top 15 down-regulated DEGs in the spheres (vs. PTM/DTM) based on the smallest logFC value in Sphere vs. PTM group (P<0.05)

**Table 9:** Top 15 DEG (DTM vs. PTM)

Gene ID	Gene Name	LogFC	*P*-value
PLXDC1	Plexin Domain Containing 1	4.402917	4.59E-32
TENM4	Teneurin Transmembrane Protein 4	2.373647	8.95E-28
ADORA2 A	Adenosine A2a Receptor	−4.38059	5.15E-20
ANKH	ANKH Inorganic Pyrophosphate Transport Regulator	1.833142	1.80E-17
GAS7	Growth Arrest Specific 7	4.026729	5.94E-16
KIAA0040	KIAA0040	2.121132	1.23E-14
PDLIM3	PDZ And LIM Domain 3	1.498271	4.72E-13
GUCY1 A3	Guanylate Cyclase 1 Soluble Subunit Alpha 1	2.291166	4.96E-12
CMKLR1	Chemerin Chemokine-Like Receptor 1	2.280106	1.60E-11
RP11-588 K22.2	RP11-588 K22.2	2.665692	3.00E-11
NPPB	Natriuretic Peptide B	−4.07455	4.81E-11
RBFOX1	RNA Binding Fox-1 Homolog 1	3.391342	5.82E-11
MDGA1	MAM Domain Containing Glycosylphosphatidylinositol Anchor 1	1.535006	6.88E-11
SERTAD4-AS1	SERTAD4 Antisense RNA 1	2.488595	1.17E-10
ZNF521	Zinc Finger Protein 521	2.88155	1.94E-10

Table 9 shows the gene ID, gene name, Log FC and *P*-value of the top 15 DEGs (DTM vs. PTM) based on the smallest *P*-value (*P*<0.05)

#### Analyses of the Potential Activated Pathways of TM Progenitor Cells

**Fig. 2:**
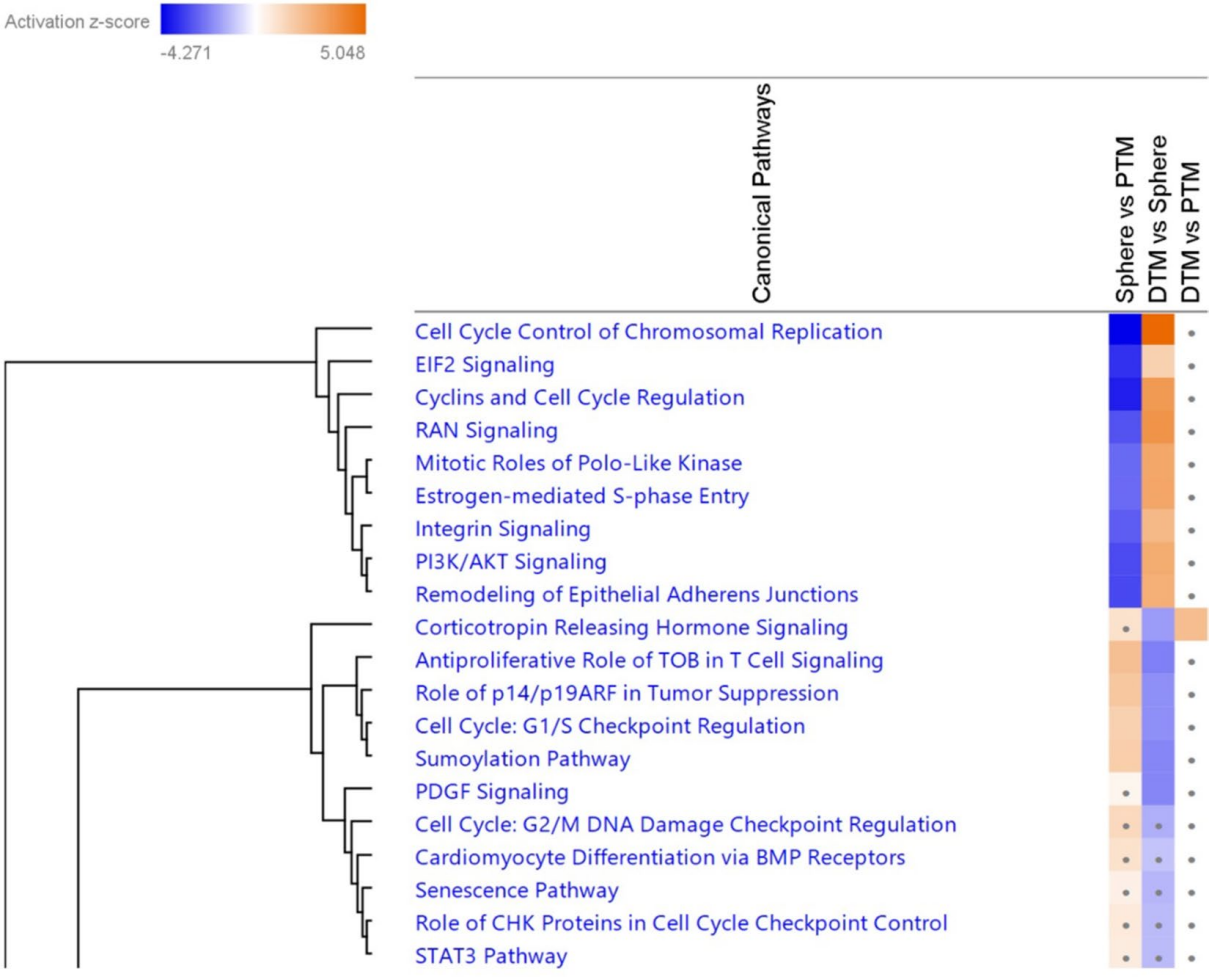
Shows heat-map of the top 20 predicted activated or inhibited canonical pathways (*P*-value < 0.05) related to the cell cycle and development in the three comparison groups by IPA analysis. Orange colour: the pathways were activated (Z score > 0); blue colour: the pathways were inhibited (Z score < 0). White colour: Z-score = 0; Grey dots: the pathways with Z-score < 1.5 and >−1.5

The potential pathways that could be activated or inhibited in PTM, TM spheres or DTM cells were analysed by the pathway analysis function of the IPA. Figure 2 showed the heatmap of the significant pathways related to cellular development and proliferation in the three comparison groups (sphere vs. PTM, DTM vs. sphere and DTM vs. PTM). The result shows that the SUMOylation pathway, Platelet-derived growth factor (PDGF) pathway and Signal transducer and activator of transcription 3 (STAT3) pathway are predicted to be activated in the TM spheres by compared with PTM and DTM cells. These pathways are associated with cell development, self-renewal and stemness maintenance of stem cells [26–29]. The whole predicted pathways in three groups were shown in the Online Resource-3.

#### Analyses of the Potential Biological Functions of the TM Progenitor Cells

**Fig. 3:**
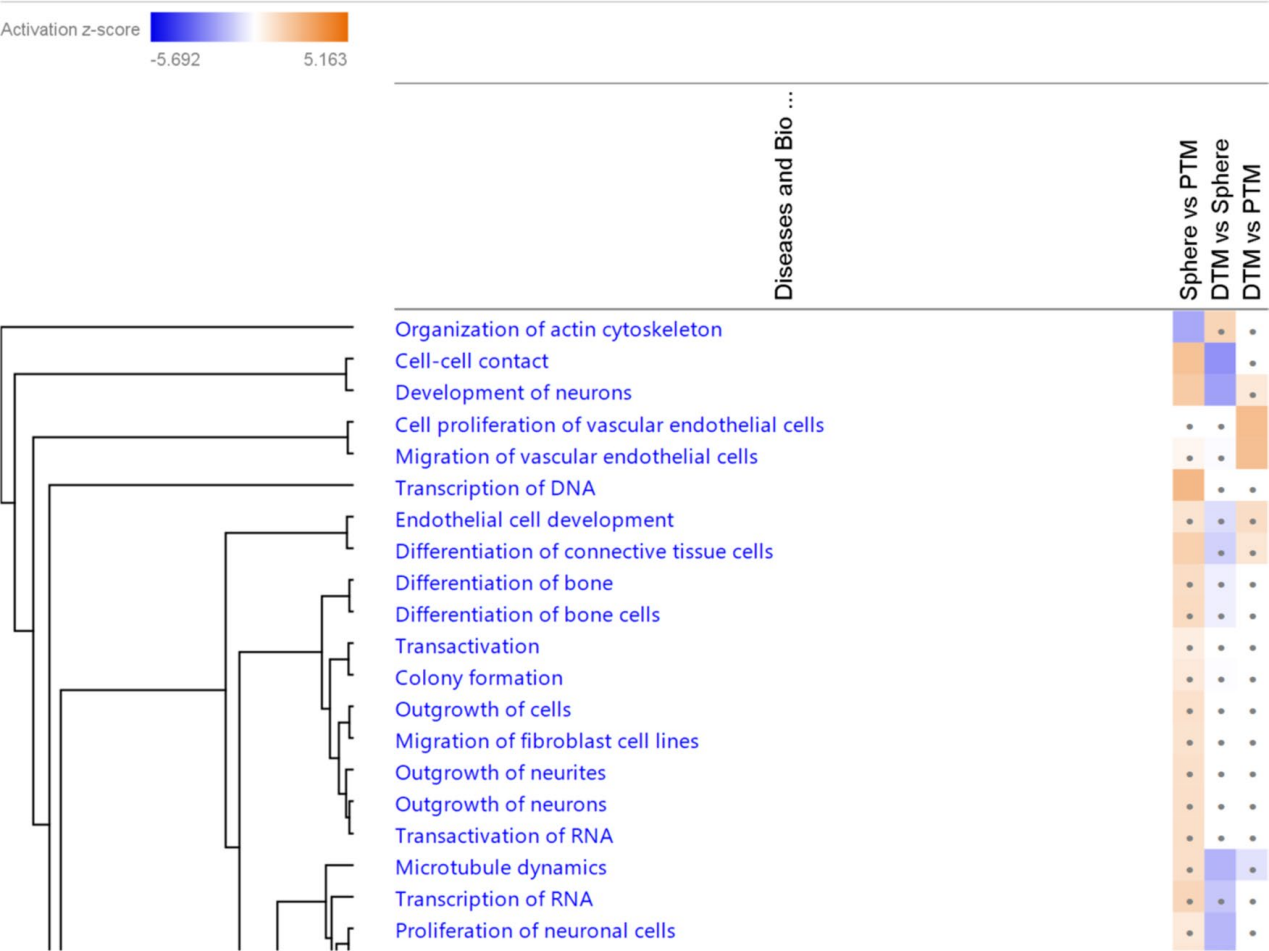
Shows heat-map of the top 20 predicted activated or inhibited biological functions related to cell development, proliferation and differentiation in the three comparison groups (*P* < 0.05). Orange colour: the biological functions were activated (Z score > 0); blue colour: the biological functions were inhibited (Z score < 0). White colour: Z-score-0; Cells with grey dots: Z-score < 1.5 and >−1.5. Cells without grey dots: Z-score > 1.5 or <−1.5

Biological function analyses of the DEGs in PTM, TM spheres and DTM cells were performed by IPA. The heatmaps of biological functions related to the cell development, proliferation and differentiation in three cell types were shown in Fig. 3. The results show that the development of neurons and development of endothelial cells were activated in the TM sphere compared with PTM and DTM cells. This finding suggests that the development related functions were activated in TMPCs and also confirms that the TM cells can be developed from the neural crest cells. Compared with the PTM and DTM cells, the S phase and G1 phase of the cell cycle in TM spheres were predicted to be inhibited. This finding may indicate the slow cycling of the TM progenitor cells. DTM cells were also shown that they might have a more vital proliferation ability than the PTM cells. The whole predicted biological function in three groups were shown in the Online Resource-4.

#### Analyses of the DEGs that may play a critical role in maintaining the un-differentiated status of the TM progenitor cells

**Fig. 4:**
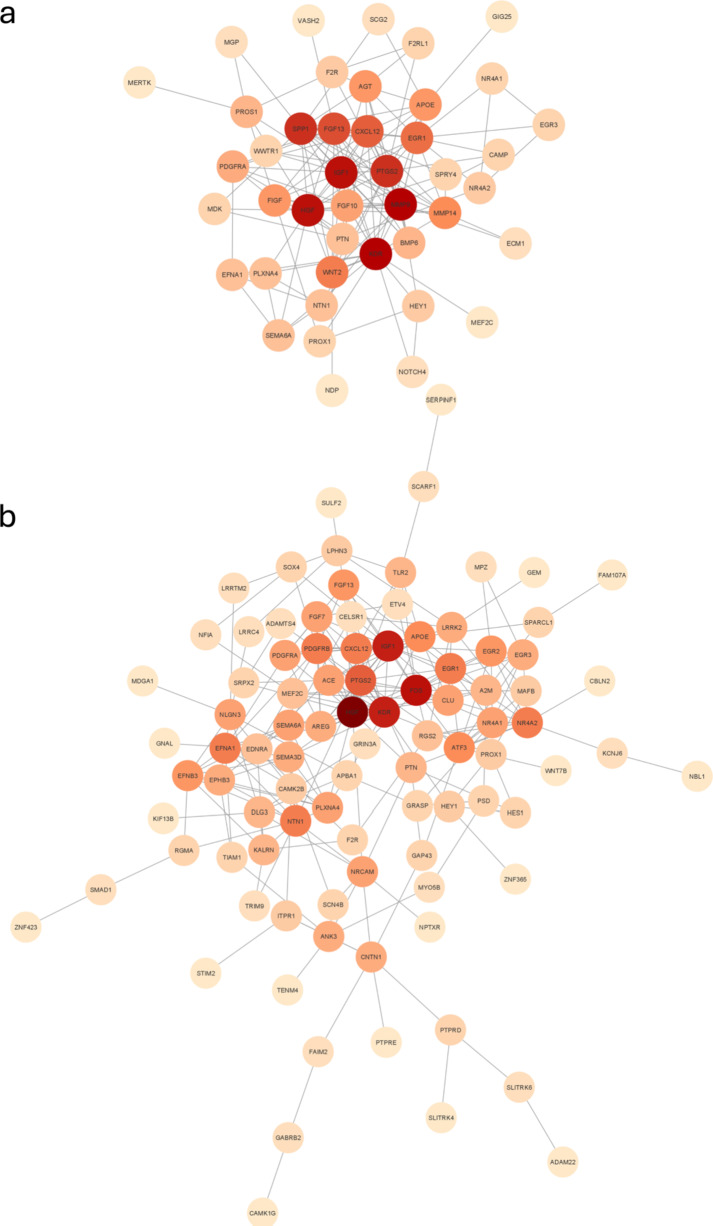
Shows protein interaction network of the DEGs related to endothelial cell development (a) or neuron development (b). The different colours mean the different levels of the node degrees; the darker colours mean the node degree is higher, which means there are more edges connected to that node

Except for the pathways and biological functions of the TM progenitor cells, the genes that may play an essential role in maintaining the undifferentiated status of the TM progenitor cells were also worth to be explored. The whole DEGs compared between TM spheres and PTM cells were analysed by IPA to find the genes related to the development of neurons and endothelial cells. The gene ID, log2 FC and *P*-values of the genes, which were related to the development of neurons and endothelial cells, were further uploaded in the String software for building the protein interaction networks (PINs). The outcome was transferred to the Cytoscape software for analysing the nodes and edges. Figure 4-a shows the protein interaction network of the DEGs related to the endothelial cell development in TM spheres. In the figure, MMP9, IGF1, HGF, KDR, PTGS2, SPP1, FGF13 and CXCL12 were found as the nodes in developing endothelial cells. Figure 4-b shows the protein interaction network of the DEGs related to the neuron development in TM spheres, HGF, KDR, FOS and IGF1 were found as the nodes in the development of neurons.

### Nanostring Analysis

The mRNA expression in the PTM, TM spheres and DTM cells (from the three same donors as the RNA-seq and two extra donors) was analysed using the Nanostring with fibrosis gene panel to confirm part of the RNA-seq data. The Nanostring data was analysed using the nSolver 4.0 software for data normalisation and DEG analyses. The normalised counts and DEGs were then used for clustering, pathway, and biological function analyses. The significant DEGs (*p* < 0.05) from the Nanostring data were then compared with the DEGs of the RNA-seq data to find the consistent DEGs (both upregulated or downregulated DEGs in the Nanostring and RNA-seq). The consistent DEGs in the RNA-seq and Nanostring data were further compared with the Nanostring-extra data (two extra donors’ sphere samples added) for DEGs confirmation.

### Clustering Analysis of the Nanostring Data

**Fig. 5:**
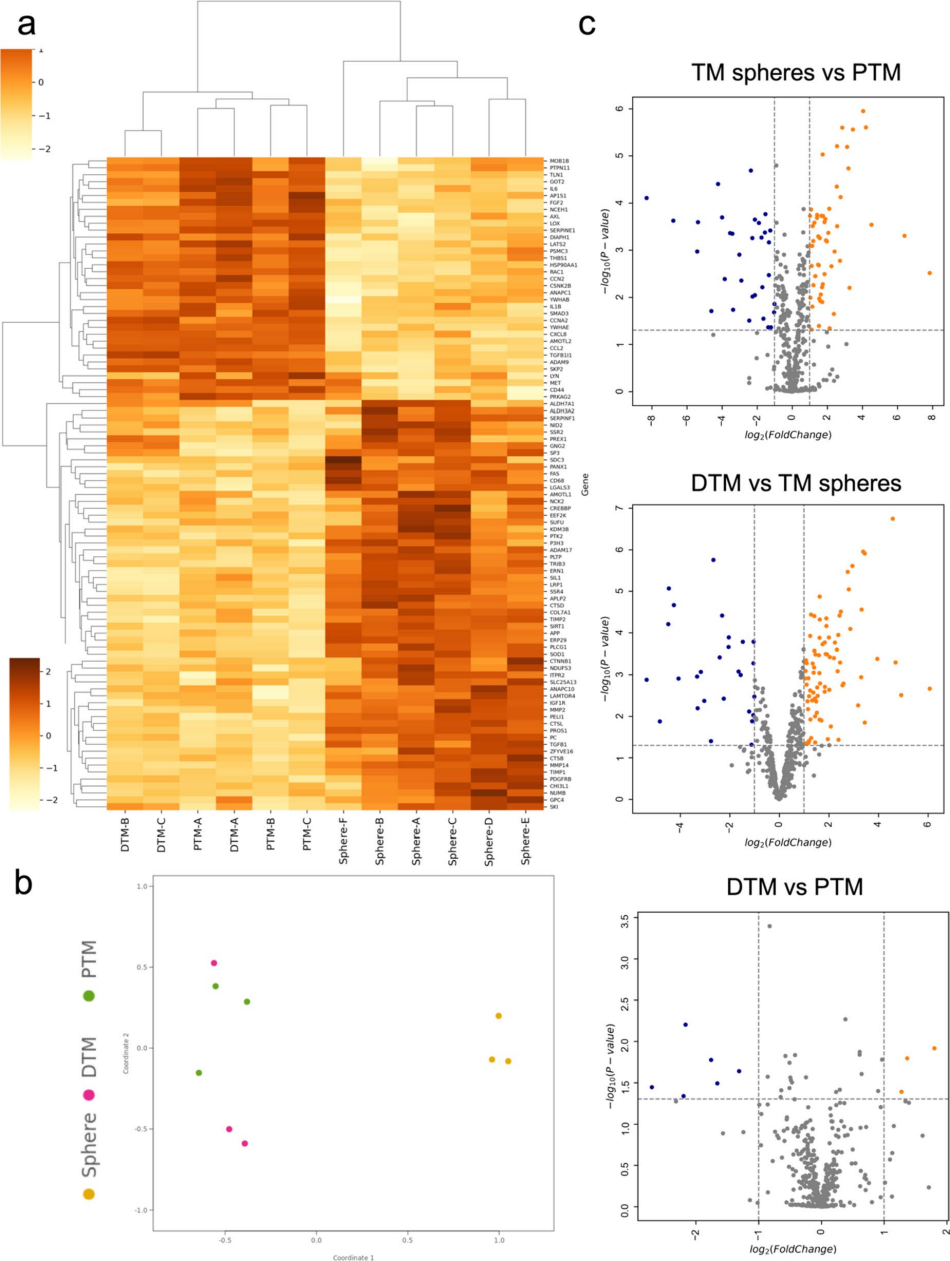
Shows visualization and clustering of the differentially expressed genes in the PTM, TM sphere and DTM cells analysed by Nanostring-fibrosis panel. **a**: Heat-map shows the top 50 significant DEGs in PTM, sphere, and DTM cells from 3 donors (Donor A-C) analysed by Nanostring. Different colours mean different Z-scores. Z-score > 0: the gene expression is upregulated; Z-score < 0: the gene expression is downregulated. *P*-value < 0.05 was considered to be statistically significant. **b**: PCA plot shows the clustering analysis of DEGs in PTM, sphere and DTM cells from 3 donors (Donor A-C) analysed by Nanostring. Yellow dots: spheres; Green dots: PTM cells; Purple dots: DTM cells. **c**: Volcano plots show the LogFC and *p*-value of the DEGs from three different comparison groups (Sphere vs. PTM; sphere vs. DTM, and DTM vs. Sphere). Orange colour: the DEGs with log 2-fold change > 1.5 and *p*-value < 0.05; Blue colour: the DEGs with log 2-fold change<- 1.5 and *p*-value < 0.05. Grey dots: the excluded genes with the log2 FC>−1 and < 1, and *P*-value > 0.05

The heatmap of the top 50 DEGs (sphere vs. PTM) in the three cell types analysed by Nanostring were shown in Fig. 5-a. The different colours mean the different Z-scores. The genes with the Z score < 0 mean the genes were upregulated. The genes with the Z score > 0 mean the genes were downregulated. The PCA plot (Fig. 5-b) showed the clustering analysis of the whole gene expression in the three cell types. The three donors’ PTM and DTM cells were shown as purple or green dots. The spheres from three donors were shown as orange dots. The PCA plot indicated that the PTM and DTM cell gene expressions were more similar compared to the TM spheres. The volcano plots (Fig. 5-c) showed the Log FC and *p*-value of the DEGs from three comparison groups (sphere vs. PTM; sphere vs. DTM, and DTM vs. sphere). The DEGs with Log FC > 1.5 or <−1.5 and *p*-value < 0.05 were considered significant. The whole DEGs in three groups from Nanostring were shown in the Online Resource-5.

#### Comparison of the DEGs from the RNA-seq and Nanostring Analyses

The significant DEGs (*p* < 0.05) of the Nanostring and RNA-seq dataset were compared using the python-automatic algorism. The LogFC values of the shared DEGs from the RNA-seq and Nanostring datasets were compared. The DEGs with the same symbols of the LogFC in the RNA-seq and Nanostring datasets were considered to be the consistent DEGs. A total of 142 DEGs (sphere vs. PTM) were found both in the RNA-seq and Nanostring datasets. 141 of the 142 DEGs were consistently upregulated or downregulated in the two datasets (consistent DEGs). One of the 142 DEGs was inconsistently expressed in the two datasets. A total of 121 DEGs (DTM vs. sphere) were found and consistently expressed in the RNA-seq and Nanostring datasets. Three DEGs (DTM vs. PTM) were found and consistently expressed in the two datasets. The top 15 consistent DEGs in three groups (sphere vs. PTM; sphere vs. DTM, and DTM vs. PTM groups) of the RNA-seq and Nanostring datasets were shown in the Tables 10, 11 and 12 (ranked by the *p*-value of the DEGs in the Nanostring dataset). The whole DEGs which were consistent in RNA-seq and Nanostring data were shown in the Online Resource-6.

**Table 10:** Top 15 DEGs (spheres vs. PTM) confirmed by RNA-seq and Nanostring

Gene	RNA-seq		Nanostring	
	LogFC	*P*-value	LogFC	*P*-value
PROS1	3.98	1.02318E-58	4.05	0.00000113
PLTP	4.16	2.42138E-34	4.21	0.0000025
TIMP1	2.87	1.48558E-26	2.86	0.00000253
MMP14	3.36	6.31658E-21	3.46	0.00000278
NID2	2.26	1.15991E-14	2.56	0.00000627
CTSL	2.95	3.99478E-34	3.13	0.00000649
ERP29	1.60	2.43327E-17	1.74	0.00000938
HSP90 AA1	−1.05	1.90367E-06	−0.904	0.0000161
TRIB3	2.92	8.25317E-41	3.21	0.0000185
AXL	−2.54	1.53198E-21	−2.35	0.0000206
CCL2	−4.43	1.03897E-16	−4.22	0.0000397
PREX1	2.97	4.46944E-07	2.55	0.0000451
PC	2.51	1.35453E-11	2.76	0.0000754
CCNA2	−4.94	2.46507E-19	−8.3	0.0000788
ERN1	2.11	5.7523E-13	2.12	0.000133

Table 10 shows the top 15 DEGs (sphere vs. PTM; the ranking is based on the lowest *P*-value in the Nanostring data) with their log FC and *P*-value in the RNA-seq and Nanostring data. LogFC<0: the DEG is downregulated; LogFC>0: the DEG is upregulated. *P*-value<0.05 is considered to be statistically significant

**Table 11:** Top 15 DEGs (DTM vs. spheres) confirmed by RNA-seq and Nanostring

Gene	RNA-seq		Nanostring	
	LogFC	*P*-value	LogFC	*P*-value
PLTP	−4.55	2.91E-39	−4.65	0.0000207
TIMP1	−2.93	2.2E-27	−3.03	0.0000458
MMP14	−3.28	2.46E-20	−3.46	0.0000592
PROS1	−3.49	8.17E-48	−3.67	0.0000645
ERP29	−1.51	9.06E-16	−1.7	0.0000712
APP	−1.20	0.000000205	−1.47	0.000125
HSP90 AA1	1.17	0.000000144	0.984	0.000227
CCL2	4.57	4.41E-18	4.32	0.000268
TRIB3	−2.56	1.15E-32	−3.03	0.000282
CCNA2	5.54	3.17E-23	8.88	0.000376
AXL	2.53	3.44E-21	2.28	0.00039
CTSL	−2.36	1.69E-23	−2.49	0.000457
ADAM17	−1.50	6.88E-13	−1.75	0.000462
ABCA1	−1.48	0.000000285	−1.75	0.000478
NID2	−1.59	4.03E-08	−1.95	0.00048

Table 11 shows the top 15 DEGs (DTM vs. sphere; the ranking is based on the lowest *P*-value in the Nanostring data) with their log FC and *P*-value in the RNA-seq and Nanostring data. LogFC<0: the DEG is downregulated; LogFC>0: the DEG is upregulated. *P*-value<0.05 is considered to be statistically significant

**Table 12:** DEGs (DTM vs. PTM) confirmed by RNA-seq and Nanostring

Gene	RNA-seq		Nanostring	
	LogFC	*P*-value	LogFC	*P*-value
LYN	−0.677432934	0.00039363	−0.823	0.000404
IL1B	−2.105035463	0.0000048	−2.16	0.00629
CASP4	−0.778375923	0.000104821	−0.854	0.0268

Table 12 shows the DEGs (DTM vs. PTM; the ranking is based on the lowest *P*-value in the Nanostring data) with their log FC and *P*-value in the RNA-seq and Nanostring data. LogFC<0: the DEG is downregulated; LogFC>0: the DEG is upregulated. *P*-value<0.05 is considered to be statistically significant

#### Validation of DEGs from RNA-Seq Analysis Using RT-PCR

The differentially expressed genes (DEGs) in PTM cells, spheres and DTM cells analysed by the RNA-seq were confirmed by RT-PCR. The DEGs were divided into three groups: the classical TM cell markers (including Matrix Gla Protein (MGP), Chitinase 3 Like 1 (CHI3L1) and Aquaporin 1 (AQP1)), the stem cell markers (including Ankyrin-G (ANKG), Nestin (NES), Notch Receptor 1 (NOTCH1), SRY-Box Transcription Factor 2 (SOX2) and Octamer-binding transcription factor 4 (OCT4)) and potential DEGs (including Secreted Protein Acidic And Cysteine Rich (SPARC), SPARC Like 1 (SPARCL1), Transgelin (TAGLN), Collagen Type IV Alpha 2 Chain (COL4 A2) and Plexin Domain Containing 1 (TEM7)).

**Fig. 6:**
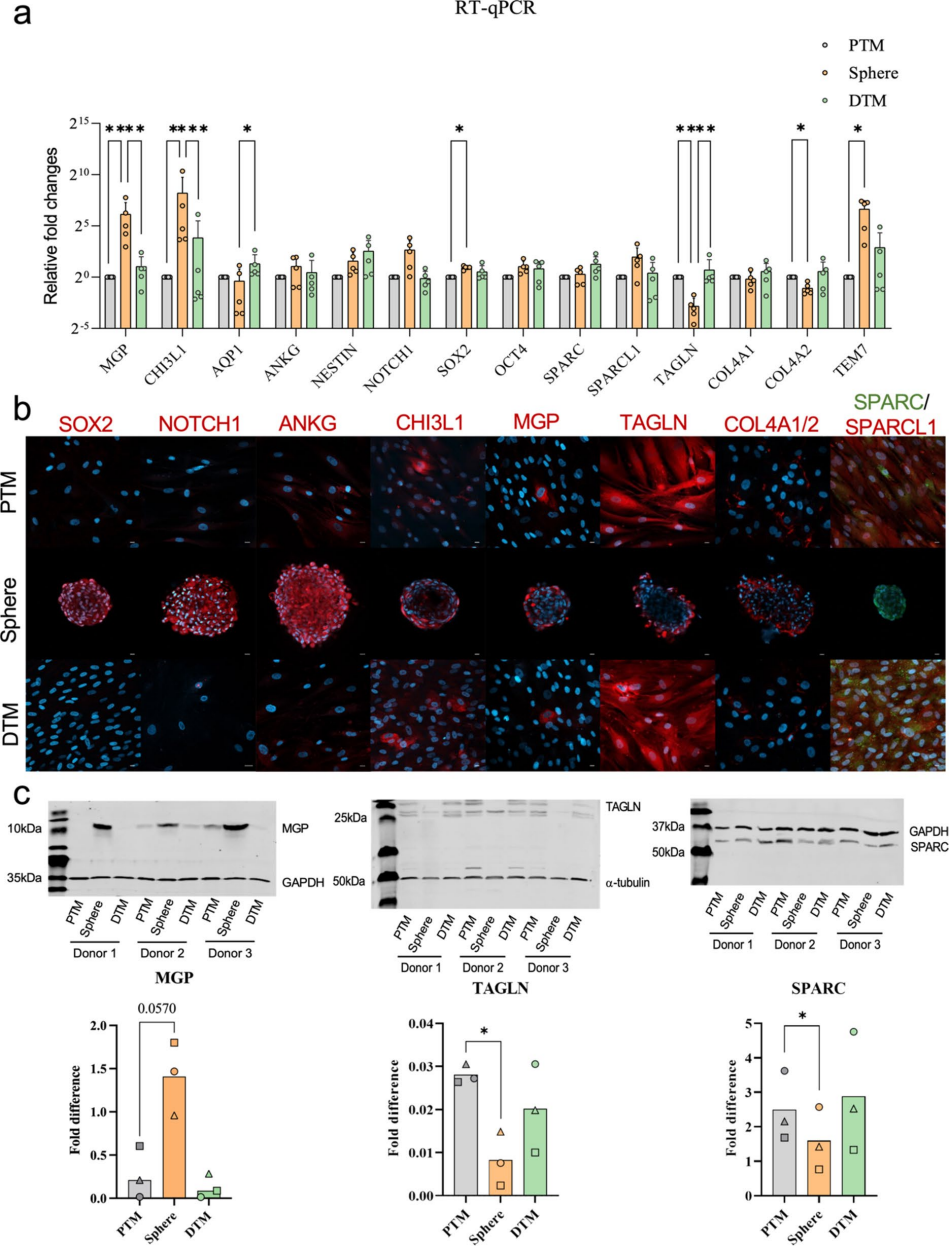
Shows **a**: RT-PCR analyses show the expression of classical TM cell markers (MGP, CHI3L1 and AQP1), stem cell markers (ANKG, NESTIN, NOTCH1, SOX2 and OCT4) and potential differentially expressed markers (SPARC, SPARCL1, TAGLN, COL4 A1, COL4 A2 and TEM7) in the PTM, TM sphere and DTM cells. *P*-value below 0.05 was considered statistically significant (*P < 0.05; **P < 0.01; ***P < 0.001). Gene expressions were normalized to GAPDH. One-way ANOVA and Friedman’s test were used in the statistical analyses. *P*-value < 0.05 was considered to be statistically significant (*P < 0.05; **P < 0.01; ***P < 0.001). **b**: Immunofluorescence staining of the classical TM cell markers, stem cell markers and potential differentially expressed markers in the PTM, sphere and DTM cells. Nucleus were stained with DAPI. **c**: Western blotting analyses show the expression of MGP, TAGLN and SPARC in the three cell types. *P*-value below 0.05 was considered statistically significant (*P < 0.05; **P < 0.01; ***P < 0.001)

The RT-qPCR analysis results showed that the SOX2 was upregulated in the TM spheres by comparing with the PTM cells (*p* = 0.0342) (Fig. 6-a). The classical TM cell markers MGP and CHI3L1 were upregulated in the spheres by comparing with the PTM (MGP: *p* = 0.007; CHI3L1: *p* = 0.0211) and DTM cells (MGP: *p* = 0.0019; CHI3L1: *p* = 0.0005). AQP1 was downregulated in the TM spheres compared with the DTM cells (*p* = 0.0324) (Fig. 6-a). The potential DEG marker TAGLN was downregulated in the spheres compared with the PTM (*p* = 0.0062) and DTM cells (*p* = 0.0068). COL4 A2 was downregulated in the spheres compared with the PTM cells (*p* = 0.0166). TEM7 was higher expressed in the spheres than in the PTM cells (*p* = 0.03) (Fig. 6-a).

## Immunocytochemical Analysis of DEGs

### Classical Stem Cell Markers

The PTM, spheres and DTM cells (*n* = 3) were stained with classical stem cell markers (SOX2, PAX6, OCT4 and nestin). The immunocytochemistry results showed that SOX2 staining was positive in TM spheres but not in the PTM and DTM cells (Fig. 6-b). Staining of PAX6 and Nestin showed in Online Resource-7.

### Classical TM Cell Markers

The PTM, spheres and DTM cells (*n* = 3) were stained with classical TM cell makers (MGP and CHI3L1). MGP and CHI3L1 staining were found positive in a few numbers of PTM, DTM cells and the edge cells of the TM spheres (Fig. 6-b).

#### Potential DEGs

The immunocytochemistry analysed the potential DEG expression (including TAGLN, COL4 A1/2, TEM7, SPARCL1 and SPARC) in the three cell types. Positive TAGLN staining was observed in all PTM and DTM cells and also found in a few cells in TM spheres (Fig. 6-b). The multiple staining of SPARC and SPARCL1 showed that SPARC and SPARCL1 were both positive in the PTM and DTM cells, and the localisation of these two markers in the cells is different. The SPARC staining was found in a few cells in the spheres, and the SPARCL1 staining is absent in the spheres (Fig. 6-b). COL4 A1/2 positive staining was found in the extracellular matrix in PTM cells, TM spheres and DTM cells (Supplementary-S8). IgG controls showed in Supplementary-S8.

### Western Blotting Analyses of the DEGs

Western blotting analyses of MGP, SPARC and TAGLN protein expression in PTM cells, TM spheres and DTM cells were showed in Fig. 6-c. MGP protein was found highly expressed in the TM spheres comparing with the PTM and DTM cells (PTM vs. Spheres: 0.057; sphere vs. DTM: *P* = 0.7397; PTM vs. DTM: *P* = 0.0978). TAGLN protein expression was decreased in TM spheres by comparing with the PTM cells (PTM vs. Sphere: *P* = 0.0267; spheres vs. PTM: *P* = 0.5006; PTM vs. DTM: *P* = 0.2886). SPARC was found decreased in TM spheres comparing with the PTM cells (PTM vs. Sphere: *P* = 0.0184; spheres vs. PTM: *P* = 0.2012; PTM vs. DTM: *P* = 0.698).

## Discussion

TMPCs have been reported to have the stem cell properties and have potential to replace the TM cell in the POAG patient. The specific markers for identifying the distinguishing the TM progenitor cells from the TM cells are still elusive. In our previous studies, we have successfully isolated and expanded the TMPCs in vitro. This study investigated the global changes in gene expression of PTM, TMPC spheres and DTM cells, which can contribute to find the specific expressed genes as well as activated pathways and biological function in TMPCs.

In this study, we identified a group of genes differentially expressed in the three cell types. Interestingly, the classical TM markers MGP and CHI3L1 genes were upregulated in the TMPCs compared to the PTM cells. MGP is a member of the family of vitamin-K2 dependent, Gla-containing proteins. It has been shown that MGP can bind to the bone morphogenetic protein-2 (BMP2; a member of the TGFβ family of growth factors) and inhibit the calcification in the ECM [30]. In 2015, Borras et al. found that the MGP gene was expressed in TM tissue, the ciliary muscle and immediate sclera [31]. In 2017, it was reported that MGP could promote endothelial differentiation in endothelial progenitor cells derived from mouse embryonic stem cells (ESCs) by inhibiting bone morphogenetic protein 4(BMP-4) [32]. BMP-4 is known as an essential factor for maintaining the stemness and multipotency of mesenchymal stem cells [33]. This evidence shows that MGP could promote the differentiation of the stem cells by inhibiting the BMP-4 expression. In our ICC results, we found that MGP was highly expressed in the edge cells of the TM spheres, which might indicate that the differentiation process was activated in those cells.

CHI3L1, also known as YKL-40, is a secreted glycoprotein encoded by the CHI3L1 gene. The biological function of CHI3L1 is still unclear, and its expression is associated with inflammation, extracellular tissue remodelling and fibrosis. CHI3L1 was reported to activate the (phosphorylates) focal adhesion kinase (FAK) together with integrin αvβ3. FAK could further activate the phosphatidylinositol 3 kinase (PI3 K)/Akt signalling pathways, which are believed critical for cell survival, proliferation and differentiation [34, 35]. CHI3L1 protein was reported to be highly expressed in the neural crest cells of human embryos by using immunohistochemistry [36]. Hoover et al. reported that CHI3L1 protein expressed in osteoblasts and chondrocytes derived from mesenchymal stem cells (MSCs). The undifferentiated MSCs were found to make CHI3L1 mRNA, but the CHI3L1 protein was not detected in MSCs [37]. CHI3L1 mRNA was also found to be highly expressed in TM progenitor spheres and TM cells by microarray analyses [38]. Our study showed that CHI3L1 was higher expressed in TMPC spheres than in TM cells by RT-PCR analyses. Like MGP, CHI3L1 was also expressed in the edge cells of the TMPC spheres. These findings suggest that CHI3L1 may play a role in regulating the survival, proliferation and differentiation of TMPCs.

In addition, we also found a group of potential genes which may play crucial roles in TMPCs, such as TEM7, MMP9, IGF1, APOE, BMP2, TIMP1, FOS and KDR. TEM7 was reported to interact with extracellular matrix components and has been linked with neuronal stem cells [39–41]. MMP9 is a member of the matrix metalloproteinase family. It was reported to play key roles in the recruitment of stem and endothelial progenitor cells from the quiescent niche in bone marrow [42] and increase the proliferation and migration of embryonic neural stem cells [43]. Insulin-Like Growth Factor 1 (IGF1) is a protein mediating growth and development. IGF1 was reported to enhance the proliferation of MSCs with lower apoptosis [44]. Its receptor IGF1R was also found to be expressed in the stem cell under conditions favourable for self-renewal [45]. APOE is an apolipoprotein that mainly functions in lipoprotein-mediated lipid transport. Recently, APOE was also shown to play key roles in regulating the development of hippocampal neurons and maintaining neural stem cells [46, 47]. BMP2, a growth factor of the TGFβ superfamily, was reported to play a critical role in many development-related physiology processes, such as cardiogenesis, neurogenesis, and osteogenesis [48]. TIMP1 is a metalloproteinase inhibitor with specific inhibition of MMP9 [49] and also play critical roles in regulating cell differentiation, migration and cell death. Lee et al. reported that TIMP1 was a key regulator of CD63 and integrin-mediated signalling, which is crucial for regulating neural stem cell adhesion and migration [50]. FOS is a protein which can regulate the TGFβ signalling by forming a multimeric complex with SMAD3/SMAD4/JUN [51]. FOS has been reported to regulate the central nervous system, immune system and bone development [52]. KDR) is a vrosine-protein kinase that acts as the cell-surface receptor for vascular endothelial growth factor A (VEGFA) [53]. KDR plays a crucial role in the regulation of embryonic hematopoiesis and vascular development [54, 55]. In this study, we found these genes were upregulated in TMPCs and were the node genes linked to the development related functions by bioinformatics analyses. These findings may indicate their crucial roles in regulating the behaviours of TMPCs.

Pathway analyses of the RNA-seq data showed that some of the pathways activated in TMPCs were associated with survival and self-renewal of the stem cells, such as the cell cycle checkpoint regulation pathways, SUMOylating pathway and STAT3 pathway.

SUMOylation pathway is a highly dynamic post-translational modification process, which was reported to play an essential role in the self-renewal of somatic cyst stem cells (CySCs) in adult Drosophila testis [56]. Reduction of SUMOylation promoted the differentiation and inhibited the proliferation of CySCs [56]. In induced pluripotent stem cells, inhibition of SUMO caused the loss of crucial pluripotency markers [57]. In this study, the SUMOylation pathway was predicted to be activated by IPA analysis with predicted activation of Death Domain Associated Protein (DAXX). DAXX is an H3.3 chaperone, which plays an essential role in the SUMOylation pathway. DAXX has been shown to have the ability to maintain the pluripotency of embryonic stem cells (ESCs) [58]. These findings suggest that the SUMOylation pathway can play a crucial role in maintaining the stemness of TMPCs.

G1/S checkpoint regulation pathway related to cell arrest was found to be activated in TMPCs. In this pathway, the cell cycle inhibitor p21 (CDKN1 A) and P27 (CDKN1B) were predicted to be activated in TMPCs by IPA analysis. CDKN1 A and CDKN1B have been shown to play crucial roles in maintaining cells arrested in the G0 phase [59, 60]. They may contribute to maintaining the quiescent state of TMPCs in vivo. In addition, the G2/M checkpoint regulation pathway was also activated. In this pathway, tumour protein P53 (TP53) was predicted to be activated in TMPCs by IPA analysis. TP53 was considered to contribute to the G2-phase arrest [61]. These findings indicate that TMPCs can be in the quiescent state, which is observed in the other adult stem cells.

STAT3 pathway is a process, which plays essential roles in cell development, proliferation, survival and carcinogenesis [62]. It was reported that the increase of STAT3 activation could convert human epiblast-derived stem cells to naive pluripotency [63]. STAT3 was shown to interact with JAK/STAT3 and Notch pathways to maintain neural precursors during the development of early neocortical in mice [64]. STAT3 can also play an essential role in the self-renewal of adult muscle satellite cells in the injured muscle [62, 65]. Our study found that the STAT3 pathway was predicted to be activated in the TMPCs, which may indicate its role in maintaining the stemness and self-renewal of the TMPCs.

By comparing the gene profile of TMPCs, PTM and DTM cells, this study found a group of biological functions in the TMPCs that differed from that in the PTM or DTM cells. These functions include the organization of actin cytoskeleton, cell-cell contact, development of neurons, development of endothelial cells, transcription of DNA and RNA, and neuronal cell proliferation. In the TMPCs, the development-related processes were predicted to be the main biological functions that differed from the PTM and DTM cells. The activation of development of neurons and endothelial cells in TMPCs can confirm that TMPCs can be developed from neural crest cells and cranial paraxial mesoderm-derived cells [66, 67]. The decreased actin cytoskeleton organization and increased cell-cell contact in TMPCs compared to PTM/DTM cells also suggested the different cell fates of these cell types. In addition, the DTM cells have increased cell proliferation and DNA/RNA transcription, which may indicate that DTM cells have higher cell viability than PTM cells. These findings may indicate that TMPCs and their differentiated cells have the potential to repopulate the TM cells in the POAG eyes.

Recently, a few studies used the single cell sequence technique to explore the different cell types and related markers in the TM tissue. In 2020, Patel et al. performed the single cell sequence analysis of the human outflow tissue and identified 12 types of cells in the outflow tissue [68]. They reported different gene expression combinations that uniquely identified each of the cell clusters (Online Resource-1-Figure-S1-a). The gene expression combinations were compared with the gene expression of the TM cells and TM progenitor cells by our RNA-seq analyses which shows in the Online Resource-1-Figure-S1-b.

Comparative analysis showed that nerve growth factor receptor (NGFR), proteolipid protein 1 (PLP1) and myelin protein zero (MPZ), which were found to be expressed in Schwann cell-like cells and/or myelinating Schwann cells in TM tissue by Patel et al., were highly expressed in the TM spheres compared with primary TM cells. Decorin (DCN) and platelet-derived growth factor receptor alpha (PDGFRA), found to be expressed in TM1, fibroblast-like cells and TM2, myofibroblast like cells by Patel et al., were also found higher expressed in the TM spheres than the primary TM cells. Transgelin (TAGLN) and Actin Alpha 2, Smooth Muscle (ACTA2), which were found in TM2, myofibroblast like cells and Smooth muscle cells by Patel et al., were found highly expressed in the primary TM cells by comparing with the TM spheres. These results suggest that the TM spheres can express many specific markers of the Schwann cell-like cells, myelinating Schwann cells, TM1, fibroblast-like cells, TM2, myofibroblast like cells, smooth muscle cells and pericytes in TM tissue, but not the markers of T/NK cells, melanocytes and epithelial cells. It may indicate that the Schwann cell-like cells, myelinating Schwann cells, TM1, fibroblast-like cells, TM2, myofibroblast like cells, smooth muscle cells and pericytes found in TM tissue can be developed from the TM progenitor cells.

In 2020, van Zyl et al. conducted a single-cell transcriptomic analysis of human anterior chamber tissue, identifying four distinct TM cell subtypes: juxtacanalicular (JCT) cells, Beam A cells, Beam B cells, and Schwalbe’s line cells—the latter being a known niche for TM progenitor cells [69]. Their study provided a comprehensive list of cell type-specific marker genes, which we compared with the DEGs identified in our RNA-seq analysis (Online Resource 1, Figure S2a), with results summarized in Online Resource 1, Figure S2b.

Our analysis revealed several notable overlaps and distinctions. Prostaglandin D2 Synthase (PTGDS), identified by van Zyl et al. as a marker of Schwalbe’s line cells, was also expressed in TM-derived spheres in our dataset, reinforcing its potential role in TM progenitor cell function. Conversely, Retinoic Acid Receptor Responder 1 (RARRES1), which van Zyl et al. found in Beam A and Beam B cells, was more highly expressed in primary TM cells than in TM spheres in our study. Additionally, van Zyl et al. reported that Schwalbe’s line cells express Carbonic Anhydrase 3 (CA3) and Mitochondria Localized Glutamic Acid Rich Protein (MGARP). While CA3 was not detected in TM spheres in our dataset, MGARP was more highly expressed in primary TM cells compared to TM spheres.

These findings suggest that while some progenitor-associated markers in TM-derived spheres overlap with known TM cell populations, others do not clearly map to a specific TM subtype. This underscores the need for further investigation, particularly using single-cell resolution techniques, to accurately define the spatial distribution of progenitor markers within the TM and elucidate their functional significance.

In addition, while our in vitro study provides robust insights into TM progenitor cell biology, it may not fully capture the in vivo complexity of the trabecular meshwork. To address this, future work will include in vivo models and functional assays to validate our findings. Moreover, expanding our donor pool and incorporating more detailed demographic data will further enhance the generalizability of our results, paving the way for translational applications.

In conclusion, this study presents a global characterisation of DEGs in primary TM cells, TMPC spheres and their differentiated cells. and a comprehensive understanding of the activated pathways and biological functions of TMPCs. The trabecular meshwork plays an essential role in regulating the IOP, which is the major and only modifiable risk factor for POAG. TM cellular loss in POAG patients is significantly increased compared to the age-matched normal people. TMPCs may have the potential to repopulate the TM cells and restore the functions of TM tissue, which may contribute to regulate the IOP and further treat POAG. Understanding the gene profile, pathways and biological functions of TMPCs may help to understand and regulate the behaviours of TMPCs in physiological or pathological conditions and to develop new therapies for POAG.

## Electronic Supplementary Material

Below is the link to the electronic supplementary material.

Supplementary file1 (PDF 1.75 MB)

Supplementary file2 (CSV 1.00 MB)

Supplementary file3 (XLS 563 KB)

Supplementary file4 (CSV 563 KB)

Supplementary file5 (PNG 550 KB)

Supplementary file6 (PNG 1.16 MB)

Supplementary file7 (PNG 382 KB)

Supplementary file8 (CSV 125 KB)

Supplementary file9 (CSV 24.9 KB)

Supplementary file10 (PDF 234 KB)

## Data Availability

The datasets generated and analyzed during this study are available from the corresponding author upon reasonable request.
