# Supplementary material for: Transcriptome Profiling of Trabecular Meshwork Progenitor Cells
Source: Stem Cell Rev Rep. 2025 May 27;21(6):1776–97. doi: 10.1007/s12015-025-10900-0 (PMC12356736; doi:10.1007/s12015-025-10900-0)
Supplement: Supplementary file 1 — Supplementary file1 (PDF 1.75 MB) [file 12015_2025_10900_MOESM1_ESM.pdf]

**Fig S1**

a

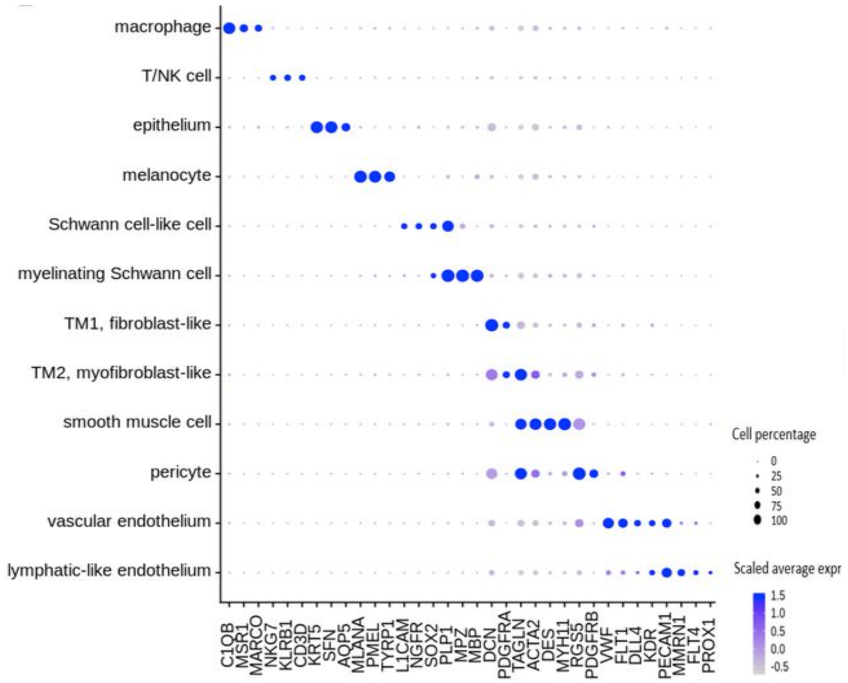


b

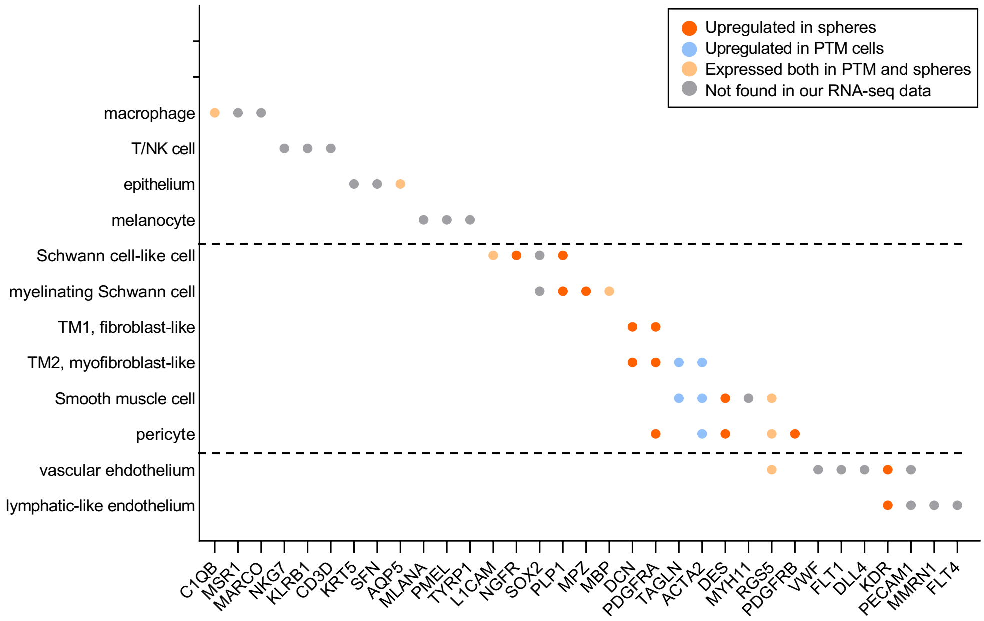


Fig S1 shows comparison dot plot of human outflow cell types [1] and the gene expression of the TM cell and TM progenitor cells by our RNA-seq analyses. **a**: Patel et al. performed the single-cell sequence analysis of the human outflow tissue and identified 12 types of cells in the outflow tissue. They reported the gene expression combinations that uniquely identified each of the cell clusters. **b**: the data are from Patel's study, and different colours represent the comparison of the data between their study and our RNA-seq analysis. The data in van Zyl's study with scaled average expression>1 and cell percentage expression>50% were used for comparison analysis. Dark orange: the genes identified by Patel et al. were found in our data

and were found upregulated in TM spheres by comparing with TM cells; Blue: the genes were found in our data and upregulated in TM cells compared with TM spheres; Light orange: the genes were found in our data, and the expression has no difference between TM spheres and TM cells; Grey: the genes were not found in our data.

**Fig S2**

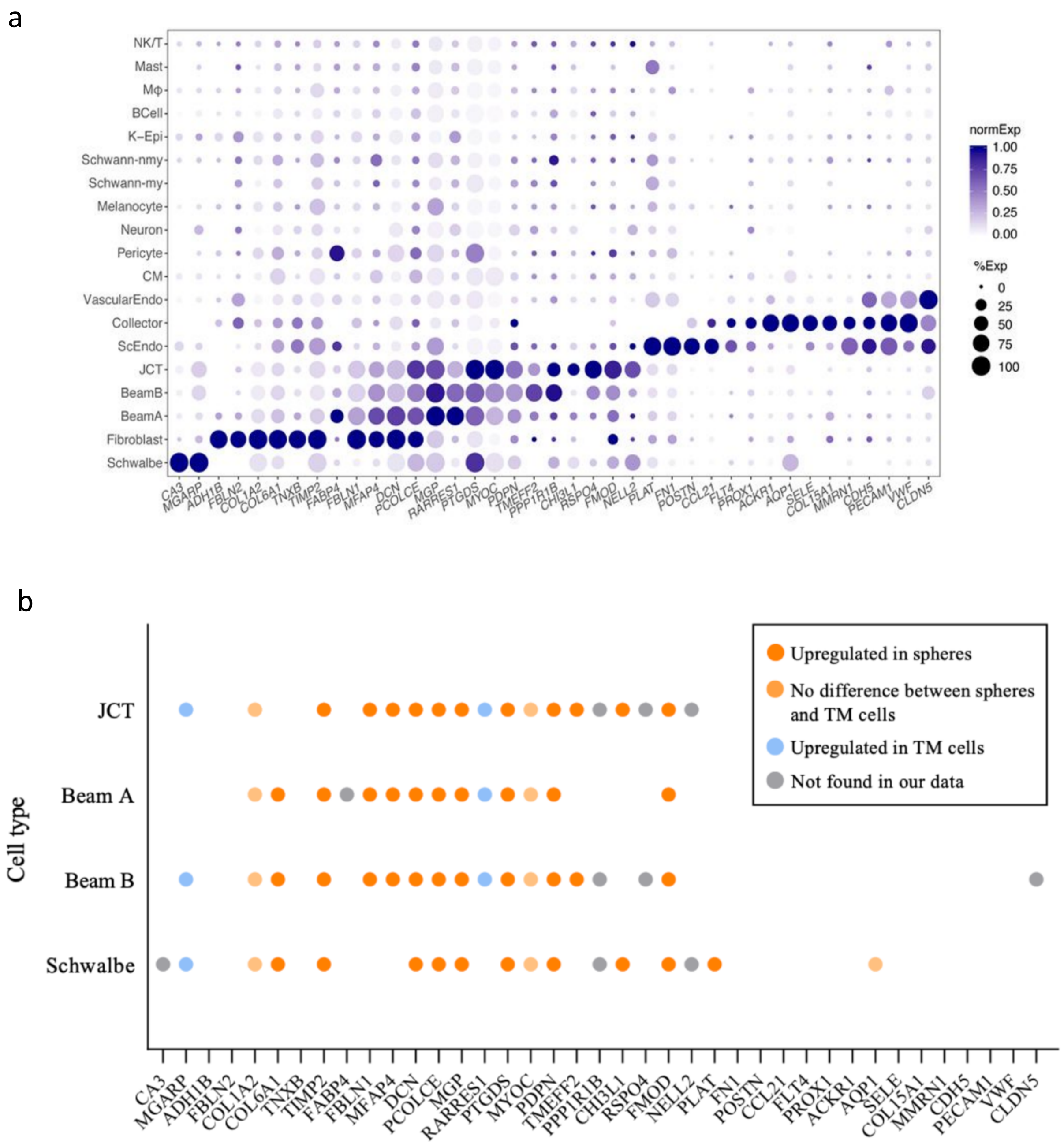


Fig S2 shows comparison dot plot of the gene expression of different TM cells type [2] and the gene expression of the TM cell and TM progenitor cells by our RNA-seq analyses. **a**: van Zyl et al. performed the single-cell sequence analysis of the human anterior chamber tissue and identified four types of cells related to TM cells. The data shown in **b** is from van Zyl's study, and different colours represent the comparison of the data between their study and our RNA-seq analysis. The data in van Zyl's study with normalized expression (normExp)>0.75 and

percentage expression (%Exp)>50% were used for comparison analysis. Dark orange: the genes identified by van Zyl et al. were found in our data and were found upregulated in TM spheres by comparing with TM cells; Blue: the genes were found in our data and upregulated in TM cells compared with TM spheres; Light orange: the genes were found in our data, and the expression has no difference between TM spheres and TM cells; Grey: the genes were not found in our data. JCT: juxtacanalicular tissue; Beam A/B: trabecular meshwork (TM) beam A/B; Schwalbe: cells in Schwalbe's line between TM and corneal endothelium (potential location of the TM progenitor cells).

1. Patel, G., et al., Molecular taxonomy of human ocular outflow tissues defined by single-cell transcriptomics. Proc Natl Acad Sci U S A, 2020. **117**(23): p. 12856-12867.
2. van Zyl, T., et al., Cell atlas of aqueous humor outflow pathways in eyes of humans and four model species provides insight into glaucoma pathogenesis. Proceedings of the National Academy of Sciences, 2020. **117**(19): p. 10339-10349.
