# Supplementary figures and images for: Transcriptome Profiling of Trabecular Meshwork Progenitor Cells
Source: Stem Cell Rev Rep. 2025 May 27;21(6):1776–97. doi: 10.1007/s12015-025-10900-0 (PMC12356736; doi:10.1007/s12015-025-10900-0)

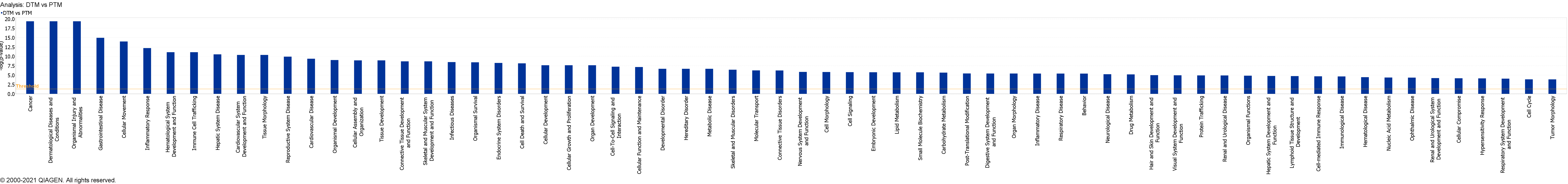

Supplement: Supplementary file 5 — Supplementary file5 (PNG 550 KB) [file 12015_2025_10900_MOESM5_ESM.png]

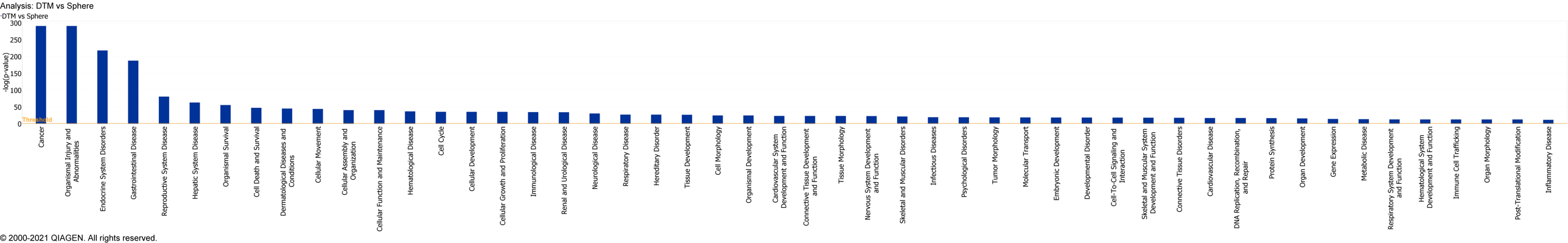

Supplement: Supplementary file 6 — Supplementary file6 (PNG 1.16 MB) [file 12015_2025_10900_MOESM6_ESM.png]

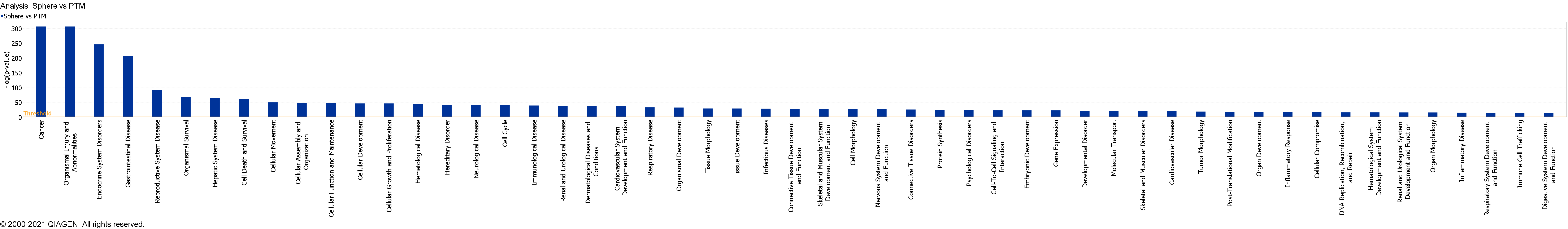

Supplement: Supplementary file 7 — Supplementary file7 (PNG 382 KB) [file 12015_2025_10900_MOESM7_ESM.png]
