# Supplementary material for: Transcriptome Profiling of Trabecular Meshwork Progenitor Cells
Source: Stem Cell Rev Rep. 2025 May 27;21(6):1776–97. doi: 10.1007/s12015-025-10900-0 (PMC12356736; doi:10.1007/s12015-025-10900-0)
Supplement: Supplementary file 10 — Supplementary file10 (PDF 234 KB) [file 12015_2025_10900_MOESM10_ESM.pdf]

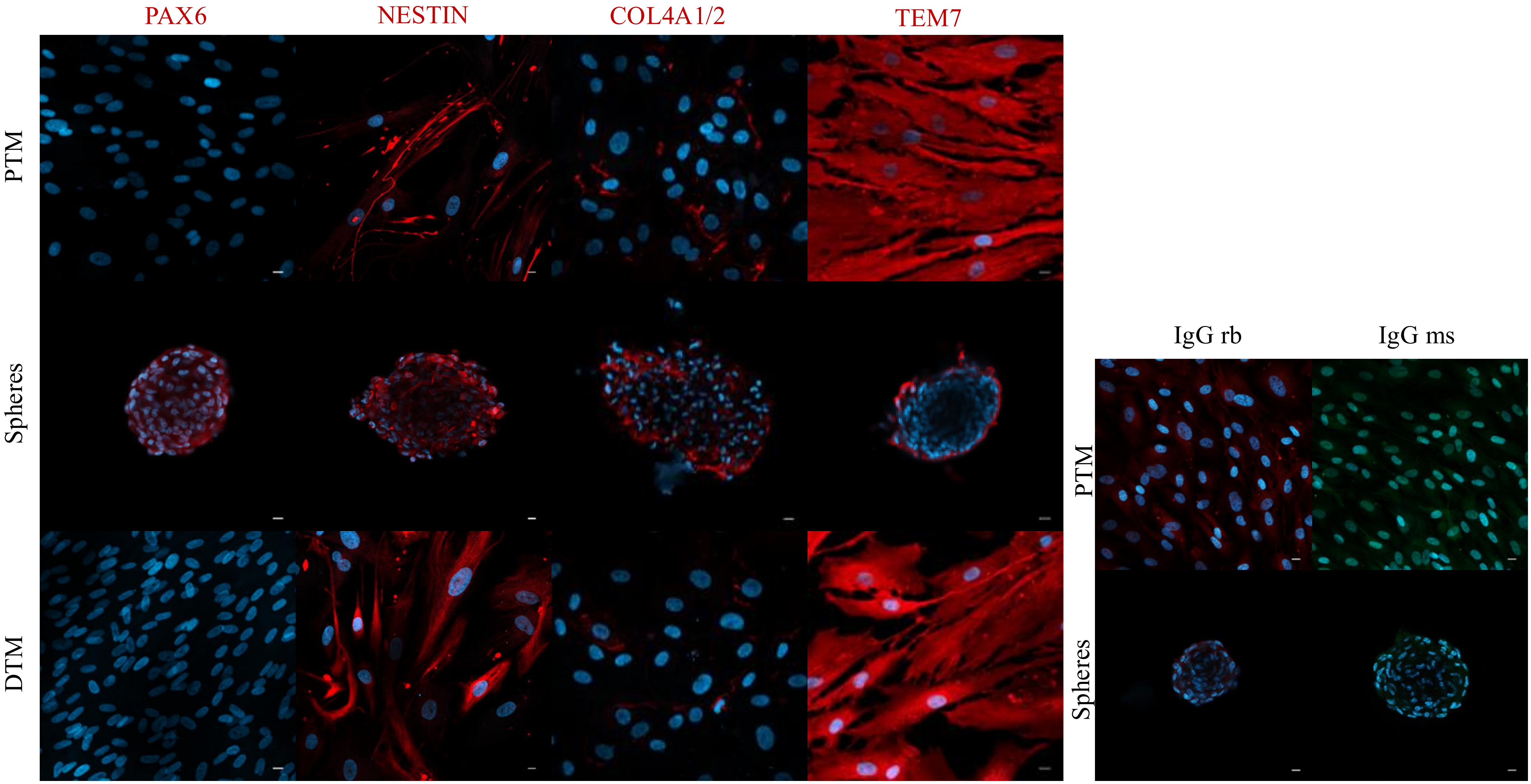

Online Resource-7 shows Immunofluorescence staining of PAX6, NESTIN, COL4A1/2 and TEM7 in the PTM, sphere and DTM cells. Nucleus were stained with DAPI.
